# Novel TiO_2_-Supported Gold Nanoflowers for Efficient Photocatalytic NO_x_ Abatement

**DOI:** 10.3390/molecules29143333

**Published:** 2024-07-16

**Authors:** Špela Slapničar, Gregor Žerjav, Janez Zavašnik, Matevž Roškarič, Matjaž Finšgar, Albin Pintar

**Affiliations:** 1 Department of Inorganic Chemistry and Technology, National Institute of Chemistry, Hajdrihova ulica 19, 1001 Ljubljana, Slovenia; spela.slapnicar@ki.si (Š.S.); gregor.zerjav@ki.si (G.Ž.); matevz.roskaric@ki.si (M.R.); 2 Gaseous Electronics, Jožef Stefan Institute, Jamova cesta 39, 1000 Ljubljana, Slovenia; janez.zavasnik@ijs.si; 3 Faculty of Chemistry and Chemical Engineering, University of Maribor, Smetanova ulica 17, 2000 Maribor, Slovenia; matjaz.finsgar@um.si

**Keywords:** heterogeneous photocatalysis, gold nanoflowers, titanate nanorods, wet impregnation, localized surface plasmon resonance effect

## Abstract

In this study, we pioneered the synthesis of nanoflower-shaped TiO_2_-supported Au photocatalysts and investigated their properties. Au nanoflowers (Au NFs) were prepared by a Na-citrate and hydroquinone-based preparation method, followed by wet impregnation of the derived Au NFs on the surface of TiO_2_ nanorods (TNR). A uniform and homogeneous distribution of Au NFs was observed in the TNR + NF(0.7) sample (lower Na-citrate concentration), while their distribution was heterogeneous in the TNR + NF(1.4) sample (higher Na-citrate concentration). The UV-Vis DR spectra revealed the size- and shape-dependent optical properties of the Au NFs, with the LSPR effect observed in the visible region. The solid-state EPR spectra showed the presence of Ti^3+^, oxygen vacancies and electron interactions with organic compounds on the catalyst surface. In the case of the TNR + NF(0.7) sample, high photocatalytic activity was observed in the H_2_-assisted reduction of NO_2_ to N_2_ at room temperature under visible-light illumination. In contrast, the TNR + NF(1.4) catalyst as well as the heat-treated samples showed no ability to reduce NO_2_ under visible light, indicating the presence of deformed Au NFs limiting the LSPR effect. These results emphasized the importance of the choice of synthesis method, as this could strongly influence the photocatalytic activity of the Au NFs.

## 1. Introduction

In this work, we have investigated the photocatalytic properties of titanium dioxide (TiO_2_) catalysts decorated with morphologically-rich gold (Au) nanoparticles in the form of nanoflowers. TiO_2_, known as a semiconducting photocatalyst, inherently exhibits the characteristic trinity of a valence band (VB), a conduction band (CB) and a band gap in between. Under the influence of light irradiation, the electrons (*e*^−^) temporarily migrate from the VB to the CB and *e*^−^–*h*^+^ pairs are formed [1]. TiO_2_ is known as a good semiconducting photocatalyst, which is inexpensive, stable in water and has low toxicity [2,3,4,5]. It occurs naturally in various crystalline phases; anatase, rutile and brookite [6]. The anatase used in this study has a wide band gap of 3.2 eV, which means that the catalyst can only be activated in the UV range of illumination [3,5,7,8]. Only 3–4% of UV light is contained in the solar spectrum [8,9]. Therefore, we need to, for instance, decorate TiO_2_ catalysts with metal nanoparticles (e.g., Au) to overcome this drawback. The Au/TiO_2_ catalyst enables the use of visible light of the solar spectrum due to the plasmonic properties of Au. Gold nanoparticles were first discovered by Faraday in 1857 and laid the foundation for the development of modern nanotechnology [10,11,12]. In 1917, Zsigmondy began the synthesis of seed-mediated growth, which is still used today. He also successfully developed an ultramicroscope that allowed him to monitor the size of gold particles. With the help of the ultramicroscope, he explained the color change of the gold solution, which was due to coagulated Au particles of different sizes [11,12,13,14]. Gold nanoparticles are known for their stability, low toxicity and activity at room temperature. They are successfully used in various fields such as catalysis [15], medicine (disease diagnosis and drugs) [16,17], energy [18,19], biology [20,21] and photocatalysis [22].

A few articles and reports have been published on the synthesis of gold nanoflowers (NFs) [23,24,25,26,27], which are widely used in medicine and biotechnology, but in the literature reviewed, we did not find any article on NFs deposited on the surface of a TiO_2_ support. We, therefore, decided to synthesize TiO_2_-decorated NF solids and investigate them in detail. The reason for developing these photocatalysts is based on an article by Arshad et al. [28], which states that the highest plasmonic effect occurs at the edges of metal nanoparticles. The shape and size of gold nanoparticles in the form of NFs depend on the amount of addition of various combinations of reducing agents. HAuCl_4_, Au-seeds and hydroquinone (HQ) are crucial reducing agents for the synthesis of NFs. As reported in the literature, different ratios of amounts lead to different colors of NFs suspensions. As the amount of added HAuCl_4_ increases, the color changes from light blue to dark blue, as the amount of added Au seeds increases, the color changes to dark blue, and as the amount of HQ increases, the color changes from reddish purple to blue [27]. The color change described occurs when one amount of the reducing agent is changed while the other two remain unchanged. The size of the gold nanoflowers decreased with the increasing addition of HAuCl_4_, and the branching of the nanoflowers also decreased, making them spherical. With increasing amounts of gold seeds, the size of NFs decreased, and with increasing amounts of hydroquinone, the size and surface branching of NFs also increased [27].

Gołąbiewska et al. synthesized and characterized TiO_2_ with the addition of Au in various forms, such as nanorods, nanospheres, and nanostars, and investigated the effects of the size and shape of the Au nanoparticles on the activity of the catalysts [29]. The UV-Vis spectrum for the TiO_2_ sample with Au nanostars showed a shift of the surface plasmon resonance to a shorter wavelength, which probably depends strongly on the defects on the TiO_2_ surface. The surface plasmon bands show a blue shift for Au nanostars, which depends on the number and length of branches/arms [29]. Gołąbiewska et al. [29] also found that the gold particles in the Au/TiO_2_ samples transform during calcination at 400 °C, which affects the photocatalytic activity, the latter depending on the shape of the gold particles. The photocatalytic activity increases as the size of the gold particles decreases. In their study, Kaur et al. compared the catalytic activity of pure TiO_2_ and Au/TiO_2_ catalysts, where the shape of Au varied (nanoparticles and nanorods). Under UV light, the addition of Au had a positive effect on the oxidation of salicylic acid, and the Au/TiO_2_ catalyst with Au in the form of nanoparticles showed the highest catalytic activity, which was due to the formation of small Au particles that were more catalytically active [30]. Au/TiO_2_ catalysts exhibit a strong visible-light absorption band that appears as a hump at 500–600 nm due to the localized surface plasmon resonance (LSPR) effect. The LSPR effect leads to the formation of “hot electrons” injected by Au nanoparticles into the CB of TiO_2_ [31,32]. The electrical contact forms a space charge layer, the Schottky barrier (SB), which traps electrons and suppresses the recombination of electrons and holes, enhancing photocatalytic activity [33,34]. The presence of SB significantly inhibits the recombination of “hot electrons” by facilitating their separation and utilization in redox processes, which plays a key role in photocatalytic reactions and photovoltaic devices. A deep understanding of the effects of Schottky barrier height (SBH) on the properties of plasmonic metal/TiO_2_ catalysts is crucial since the photocatalytic activity of catalysts containing plasmonic metals is affected by the transfer of “hot electrons” from the plasmonic metal to the conduction band of the TiO_2_ semiconductor [28].

Anthropogenic activities in recent decades have resulted in high emissions of nitrogen oxides (NO_x_) into the atmosphere, which can cause various environmental problems such as the formation of acid rain, photochemical smog, the greenhouse effect, etc. [35]. In addition, NO_x_ can react in the atmosphere with other air pollutants (volatile organic compounds (VOCs)) to form products (e.g., nitrous acid, peroxyacyl nitrate (PANI), etc.) that are even more harmful to animal, human and environmental health than NO_x_ [36]. One of the methods for removing NO_x_ is selective catalytic reduction (SCR), in which various reducing agents (e.g., H_2_, CO, NH_3_, HC, etc.) are added in such a way that NO_x_ reduction takes place at temperatures between 300 and 400 °C [37]. The advantage of using H_2_ as a reducing agent is that it can reduce NO_x_ at relatively low temperatures and produce low emissions of greenhouse gases [38,39,40]. Žerjav et al. [41] demonstrated that H_2_-assisted NO_x_ oxidation can also take place at lower temperatures (below 100 °C) by using plasmonic metal/TiO_2_ catalysts in a photothermal catalytic process, a hybrid technology that synergistically combines the advantages of thermal and photo-based catalytic approaches.

Much has been published on catalysts with TiO_2_ and Au, changing the loading of Au [42,43,44] and the size of the Au particles [45,46,47], using different methods of Au deposition on the surface of TiO_2_ [48] and various crystalline phases of TiO_2_ [49,50,51]. In our research, we focused on changing the shape of the gold. However, to our knowledge, only a few articles have been published on TiO_2_ and non-spherical gold, which exhibit unique catalytic properties [52]. The aim of this work was to successfully synthesize Au nanoflowers and decorate TiO_2_ with them for the first time. The morphological, surface, optical, and electronic properties of the obtained catalysts were studied in detail using various characterization techniques to systematically explore the influence of the plasmonic metal form on the properties of the Au/TiO_2_ catalysts. The visible-light-triggered photocatalytic activity of the synthesized catalysts was investigated for the reduction of NO_x_ in the presence of H_2_ as a reducing agent to conceptually demonstrate an alternative approach to NO_x_ reduction at room temperature. The two synthesized catalysts were also heated to explore the influence of morphology changes on the photocatalytic performance.

## 2. Results and Discussion

### 2.1. NFs suspension Characterization

**Figure 1:**
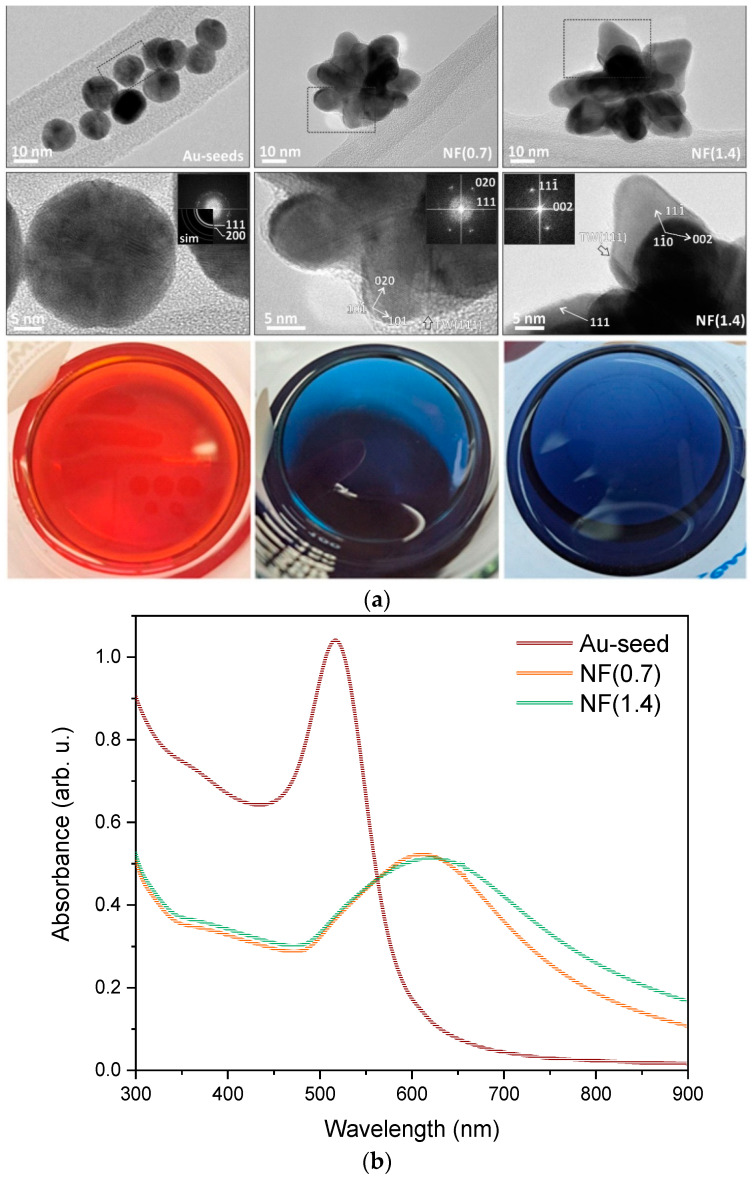
(**a**) Overview- and HR-TEM micrographs of Au particles, with corresponding FFT (Fast Fourier transform) patterns indexed for Au; the twin boundaries (TW) are marked with arrows. Photos of prepared solutions containing corresponding Au particles. (**b**) UV-Vis absorption spectra of liquid-phase samples.

The main building blocks for the synthesis of Au NFs are Au seeds, the solution of which is colored red. The prepared suspensions of Au NFs are colored blue, with the shade of blue changing slightly (Figure 1). The difference between them is due to the different amounts of Na-citrate added. The sample with less added Na-citrate (0.7 mL) is labeled NF(0.7), and the sample with more added Na-citrate (1.4 mL) is labeled NF(1.4). Figure 1a also shows the TEM and HR-TEM micrographs of the analyzed liquid-phase samples, on which the successful synthesis of the Au NFs can be seen, as SEM analysis is not a suitable method due to the nanoscale particles (Figure S1).

Au seeds are a near-spherical shape, and are polycrystalline; hence, calculated FFT pattern forms rings. The individual crystalline domains are ~5 nm. The Au nanoparticles in the NF(0.7) sample have 3D morphology, with crystalline domains ~10 nm in size. In the NF(1.4) sample, the individual segments are even larger, and the crystalline domains are ~15–20 nm. In the case of both NF samples, there are several characteristic (111) twin boundaries (TW) observed in the central part, as marked on the TEM micrographs.

The UV-Vis absorption spectra were first measured for the gold seed solution and the suspension of Au NFs. The measured spectra (Figure 1b) show that the shape of the spectrum depends on the size and shape of the gold nanoparticles. The UV-Vis spectra of NF(0.7) and NF(1.4) suspensions look very similar; minor deviations between the curves probably occur due to a change in the size and shape of the gold nanoparticles between the two samples.

The average size of NFs in the NF(0.7) solution was 32 nm and 35 nm in the NF(1.4) solution. For each sample, at least 80 particles were measured in the solution using TEM micrographs. According to Chen et al. [53], smaller nanoflowers are formed when a larger amount of Na-citrate is used in the suspension.

### 2.2. Catalyst Characterization

The aim of this work was to synthesize TiO_2_ in the form of nanorods that are decorated with morphologically-rich Au nanoparticles (nanoflowers) to increase their active surface. The synthesized catalysts are shown in Figure S2.

#### 2.2.1. TEM, SEM-EDXS, XRD and N_2_ Physisorption Analyses

**Figure 2:**
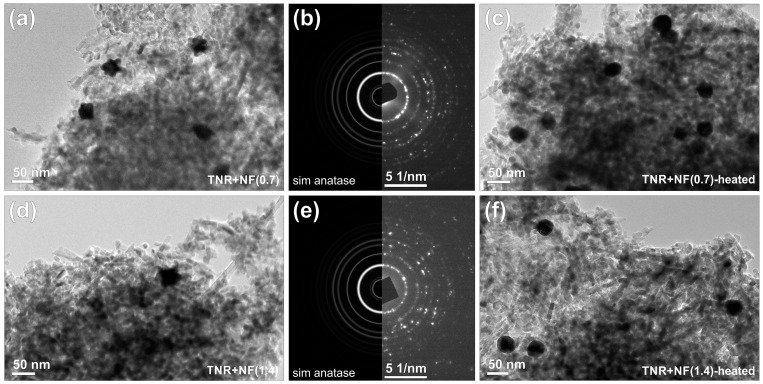
(**a**) TEM micrograph of sample TNR + NF(0.7), with (**b**) experimental SAED pattern and ab-initio simulation for anatase TiO_2_, and (**c**) TEM micrograph of sample TNR + NF(0.7, heated). (**d**) TEM micrograph of sample TNR + NF(1.4), with (**e**) experimental SAED pattern and ab-initio simulation for anatase TiO_2_, and (**f**) TEM micrograph of sample TNR + NF(1.4, heated).

The morphology of the Au/TiO_2_ catalysts was analyzed by TEM (Figure 2). The TiO_2_ is in the form of single-crystal anatase nanorods 70 nm long and 8 nm wide (see selected area electron diffraction (SAED) pattern in Figure 2). It is also evident from Figure 2a,d that the synthesis of Au/TiO_2_ catalysts was successful, as the gold ensembles are polycrystalline and have a well-defined, structured 3D shape (Figure S3). From the TEM micrographs, also shown in Figure 2, it can be seen that the deposition of Au nanoflowers on the surface of TNR is uniformly homogeneously distributed in the TNR + NF(0.7) sample, while in the TNR + NF(1.4) sample, the deposition of NFs is heterogeneous, which was already observed during the synthesis of the sample, as the sample turned darker in the upper layer and brighter in the lower layer during evaporation on the rotary evaporator (Figure S4). Figure 2 also shows the TEM micrograph of samples after heating to 300 °C, i.e., TNR + NF(0.7, heated) and TNR + NF(1.4, heated). The morphology of gold NFs changed to spheres after heating to 300 °C, which is due to the melting of the NF center at this temperature [29]. The size of the Au NFs changes minimally in both samples. Some larger aggregates are observed in the sample TNR + NF(1.4), which is due to particle agglomeration.

**Figure 3:**
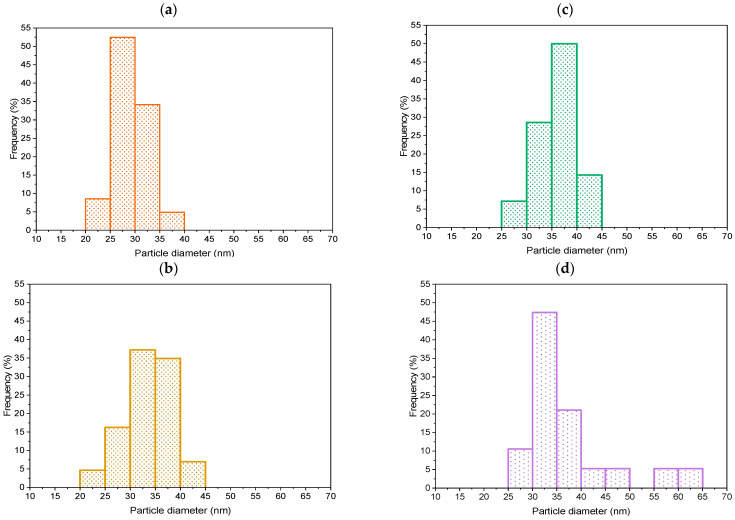
The particle size distribution diagrams of Au particles on the surface of (**a**) TNR + NF(0.7), (**b**) TNR + NF(0.7, heated), (**c**) TNR + NF(1.4), and (**d**) TNR + NF(1.4, heated) samples.

The size of the Au particles was measured considering their Feret diameter [54] measured on TEM micrographs. At least 80 Au particles were measured for each sample, the results were statistically analyzed, and a diagram of the particle size distribution was constructed. Figure 3 shows the Au particle size distribution of all investigated samples, and the average size of Au NFs in Au/TiO_2_ catalysts is listed in Table 1. The size of Au NFs did not change during the wet impregnation technique, indicating that the Au ensembles were stable. Agglomerated Au ensembles are also seen in the TEM analysis (Figure S5a). The heterogeneity of the sample TNR + NF(1.4) is also evidenced by SEM-EDXS (Table S1) and UV-Vis DR measurements (Figure S5b). The reason for the heterogeneity of this sample in terms of size and distribution of Au ensembles is the amount of Na-citrate added, since a larger amount of Na-citrate results in the formation of a larger amount of gold nuclei, which in turn results in the formation of smaller particles with higher mobility. It follows that smaller gold nanoparticles can aggregate more easily. Another possibility is that the Na-citrate residues do not react and cause the agglomeration process [55,56]. Overall, the sizes of the gold ensembles are adequate to produce an improved LSPR effect [57].

The results of the chemical analyses by EDXS and ICP-OES showed that the actual Au loading of the synthesized catalysts was close to the nominal values of 1.0 wt.% Au loading (Table 2). The sample TNR + NF(0.7) showed a homogeneous distribution of elements, while in the sample TNR + NF(1.4) the loading of Au was heterogeneous throughout the sample (Table S1). The top layer of the sample had a loading of more than 1.0 wt.%, while the bottom layer had a lower loading, which we already predicted from the color difference (Figure S4). After heating the TNR + NF(0.7, heated) and TNR + NF(1.4, heated) samples to 300 °C, the gold loading was also close to 1.0 wt.%. It follows that the composition of the sample has not changed at all, but only the shape of the gold particles has changed.

**Figure 4:**
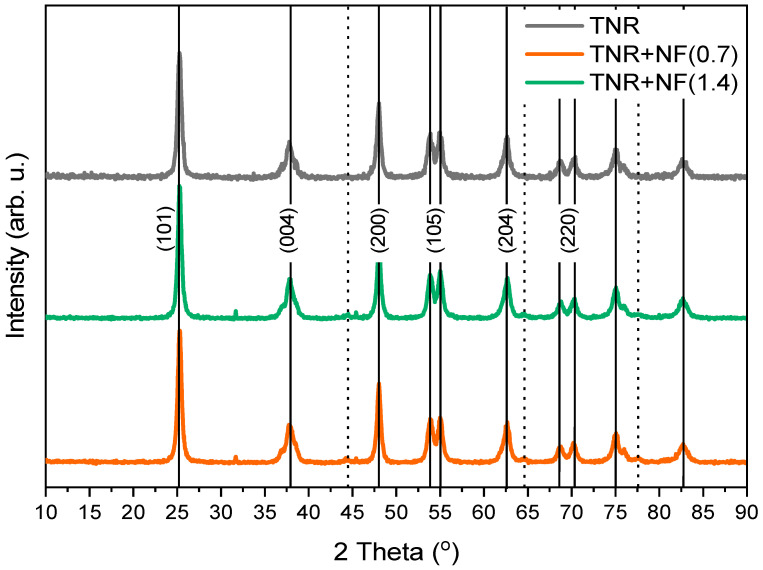
XRD diffractograms of the TNR support and catalysts containing 1.0 wt.% of Au loading. The solid vertical lines belong to anatase TiO_2_ (JCPDS 00-021-1272) and the dotted vertical lines belong to Au (JCPDS 01-1174). The apparent anatase crystallite sizes were calculated from XRD data using the Scherrer equation and are listed in Table 3.

Based on the position of the peaks in the XRD diffractograms, we can conclude that TiO_2_ is present only in the anatase form (Figure 4). Since the Scherrer equation is used to calculate the crystallite size of spherical particles (by calculating the volume of the individual crystallite) and TiO_2_ is in the form of nanorods, we can only calculate the apparent size of TiO_2_ crystallites using the XRD technique. The results of the calculations are listed in Table 3. The average apparent size of TiO_2_ crystallites is 17 nm in TNR and Au/TiO_2_ samples calculated from the anatase TiO_2_(101) diffraction peak at 25°. The apparent size of the anatase crystallites was also the same for the Au/TiO_2_ catalysts, indicating that the TNR support was not affected by the Au deposition process. In the TNR + NF(0.7) and TNR + NF(1.4) samples, the peak at 32° appears, which is not visible in the TNR sample. The peak at 32° is characteristic of the compound Na_2_O (from Na-citrate) [58].

**Figure 5:**
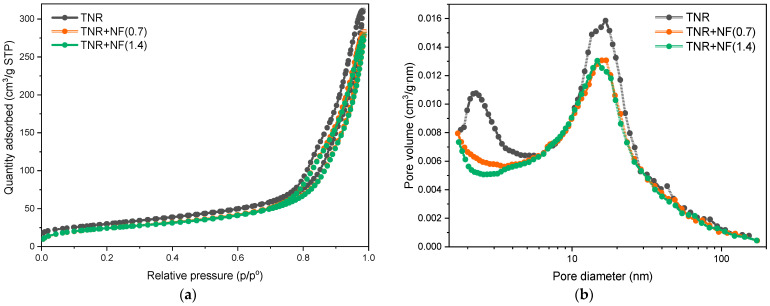
(**a**) Nitrogen adsorption/desorption isotherms of the TNR support, and TNR + NF(0.7) and TNR + NF(1.4) catalysts; (**b**) corresponding BJH pore size distribution.

The N_2_ physisorption results shown in Figure 5 and Table 3 provide information on the specific surface area (*S*_BET_), pore volume (*V*_pore_), and pore diameter (*d*_pore_) of the catalysts studied. A slight decrease in *S*_BET_ value was observed after gold was deposited onto the TNR, indicating that the pores of the TNR support were slightly clogged. From the results of the N_2_ physisorption measurements, no major differences between the values of *V*_pore_ and *d*_pore_ can be observed. Figure 5a shows the N_2_ adsorption-desorption isotherms for TNR catalysts with the addition of Au NFs. The prepared catalysts exhibit N_2_ adsorption-desorption isotherms corresponding to the type IV isotherm according to the IUPAC classification [59], which makes them belong to the mesoporous materials with pore sizes from 2 to 50 nm. The shape of the curves in Figure 5a is a type H3 hysteresis curve associated with the phenomenon of pore condensation. Again, the way the curves approach P/P_0_ = 1 shows that the materials also contain macropores larger than 50 nm. The position of the curves does not change significantly when Au NFs are used, and we thus confirm that the use of Au NFs does not affect the morphology of the catalysts. Figure 5b shows the derived pore size distribution for all catalysts. The pore diameters are in a range between 1 and almost 100 nm, with a maximum between 10 and 20 nm. It is now evident that the decrease of the *S*_BET_ in the Au decorated samples is due to the pore blockage of smaller mesopores (~1.5 nm) as observed with the disappearance of this peak in Figure 5b.

#### 2.2.2. ATR-FTIR, CO-DRIFTS and XPS Analyses

**Figure 6:**
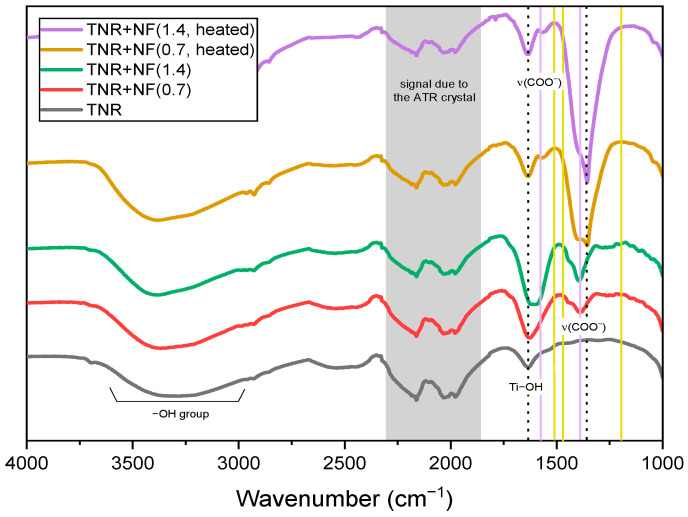
ATR-FTIR spectra of the catalysts studied. The yellow lines mark characteristic HQ peaks, the purple lines stand for characteristic Na-citrate vibrations and the dotted lines are for the peaks of the Au/TiO_2_ samples.

The ATR-FTIR spectra of the synthesized Au/TiO_2_ catalysts are shown in Figure 6. In addition, the ATR-FTIR spectra of pure Na-citrate and HQ used for the preparation of Au NFs and the ATR-FTIR spectra of the TNR support with adsorbed Na-citrate and/or HQ (without Au) are shown in Figure S6. HQ (Figure S6a) shows some prominent peaks at wavenumbers 1511 and 1465 cm^−1^ (belonging to the C-C vibrational bands of the aromatic ring), at 1350 cm^−1^ (associated with the O-H bending) and at 1192 cm^−1^ (attributed to the in-plane C-H bending) [60]. The HQ molecule could be bound to the surface of the TNR support perpendicularly via one of the OH groups or planarly via the benzene ring. As can be seen in Figure S6b, the latter binding does not allow many vibrations, so that the peaks are of very low intensity. Pure Na-citrate shows main vibrations at 1576 and 1386 cm^−1^ (Figure S6a), which belong to the asymmetric and symmetric stretching of the carboxyl groups, respectively [61,62]. After the adsorption of Na-citrate on the surface of TNR, there was no shift in the peaks (Figure S6b). The same applies to heating this material to 300 °C. In this case, only the intensity of the peaks decreased, which is due to the partial removal of Na-citrate from the TNR surface. When both Na-citrate and HQ were adsorbed on the TNR surface, the most frequent peaks belonged to Na-citrate. When both species are adsorbed, it is obvious that the extent of HQ adsorption on the TNR surface increases over one of the OH groups (the appearance of a broad peak at 1240 cm^−1^). From the recorded ATR-FTIR spectra of the Au/TiO_2_ samples (Figure 6), the most abundant peaks belong to the adsorbed Na-citrate. However, the peaks at 1576 and 1395 cm^−1^ are shifted to 1638 and 1356 cm^−1^, respectively, which we attribute to interactions of the Au NPs with the carboxyl groups in the adsorbed Na-citrate. This was confirmed by XPS analysis (see below) and is in agreement with literature reports [63]. The decrease in the intensity of the peaks at 1638 cm^−1^ and the increase in the intensity of the peaks at 1356 cm^−1^ upon heating the Au/TiO_2_ samples to 300 °C can be attributed to (i) the partial removal of the adsorbed organic compounds on the catalyst surface (Table 2) and (ii) the simultaneous rearrangement of the surface-bound C-containing species.

In addition, CO chemisorption on Au NPs was investigated by DRIFTS analysis. The results obtained, which are consistent with the findings of the TEM analysis, are presented and discussed in the Supplementary Information (Figure S7).

**Figure 7:**
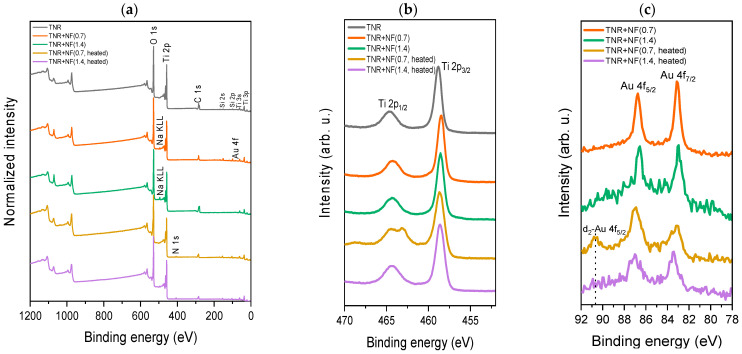
(**a**) Survey spectra and (**b**) high-resolution Ti 2p spectra for TNR, TNR + NF(0.7), TNR + NF(1.4), TNR + NF(0.7, heated) and TNR + NF(1.4, heated) samples. (**c**) High-resolution Au 4f spectra for TNR + NF(0.7), TNR + NF(1.4), TNR + NF(0.7, heated) and TNR + NF(1.4, heated) samples.

The XPS analyses are shown in Figure 7 and Figure S8. All survey spectra show intense O 1s and Ti 2p peaks and less intense C 1s peaks (Figure 7a). Ti- and O-related XPS signals originate from TiO_2_, while C 1s signal originates from adventitious carbonaceous species adsorbed on the surface of the samples during sample handling and transport to the spectrometer, as well as from adsorbed Na-citrate and HQ used in the catalyst synthesis. As the Au content was low, the Au-related peaks were also non-intense. Survey spectra also show a less intense peak for Na 1s and the XPS-excited Auger Na KLL peak, which were only present for the samples TNR + NF(0.7) and TNR + NF(1.4). The intensity of the Na 1s peak is higher for the TNR + NF(1.4) sample, as the amount of Na (from Na-citrate) added to the sample was also higher. After heating, the Na 1s peak and XPS-excited Auger Na KLL peak intensities decreased as some Na was removed from these samples. The signal of Cl was not detected in the survey spectra. Therefore, the surface concentration of Cl was below the detection limit or was not present on the surface. The latter means that the HAuCl_4_ precursor has successfully decomposed during the catalyst preparation procedure. For the samples TNR + NF(0.7, heated) and TNR + NF(1.4, heated), a N 1s peak is visible in Figure 7a, which is probably due to the preparation procedure as the samples were heated to 300 °C in the photocatalytic reactor. Figure 7b shows Ti 2p spectra (peak doublets) with Ti 2p_3/2_ peak at more negative binding energy and Ti 2p_1/2_ peak at more positive binding energy. The Ti 2p_3/2_ peak for the TNR sample is located at 458.7 eV, corresponding to TiO_2_ [60]. The Ti 2p_3/2_ peaks for the TNR Au-containing samples are located at approximately 0.3 eV more negative binding energy. The latter could suggest that the Au/TiO_2_ samples are partially reduced, with the formation of Ti^3+^. Au was confirmed for all NFs samples, where the Au 4f peaks’ intensity increases with the increase in Au content in the samples (Figure 7c). The Au 4f_7/2_ peaks in all spectra were located at approximately 83.4 eV, which is at more negative binding energy than the expected position of 84.0 eV for bulk Au (fitted spectra in Figure S8b). According to earlier reports, the peak shift toward more negative binding energies could result from the nanoparticles’ transfer of electrons from the carrier [64]. However, none of the analyzed Au-containing samples show a change in the oxidation state of Au, suggesting that Au is in the metallic state (Au was not oxidized). Figure 7c shows an additional spectral feature at 91.5 eV for the TNR + NF(0.7, heated) sample. This feature most likely corresponds to the Au 4f_5/2_ of the second doublet. The Au 4f_7/2_ peak of this second doublet is most likely located at the same position as the Au 4f_5/2_ peak of the first Au 4f doublet since this spectral feature is more intense than that for Au 4f_7/2_ of the first Au 4f doublet (the intensity ratio of the Au 4f_7/2_:Au 4f_5/2_ should be 4:3 and therefore Au 4f_7/2_ should be more intense than Au 4f_5/2_). The presence of the second Au 4f doublet could be attributed to the presence of Au particles in a different environment on the catalyst surface [65]. The high-resolution C 1s spectra are shown in Figure S8a. The C 1s spectrum for the TNR support shows a low-intensity peak at 288.8 eV which most likely originates from COO/COOH [66]. This peak can also be assigned to the COO-Au connection for the samples with 1.0 wt.% Au loading and indicates an interaction of the Au particles with Na-citrate adsorbed on the catalyst surface [67]. Moreover, Figure S8a also shows a spectral feature at about 280 eV for the TNR, TNR + NF(0.7) and TNR + NF(1.4) samples, which most likely originates from impurities during the synthesis of the TNR support and could be assigned to states with pπ-like (C 2p–O 2p) bonding character from O lone pairs [68,69]. This peak disappears after heating the samples to 300 °C.

**Figure 8:**
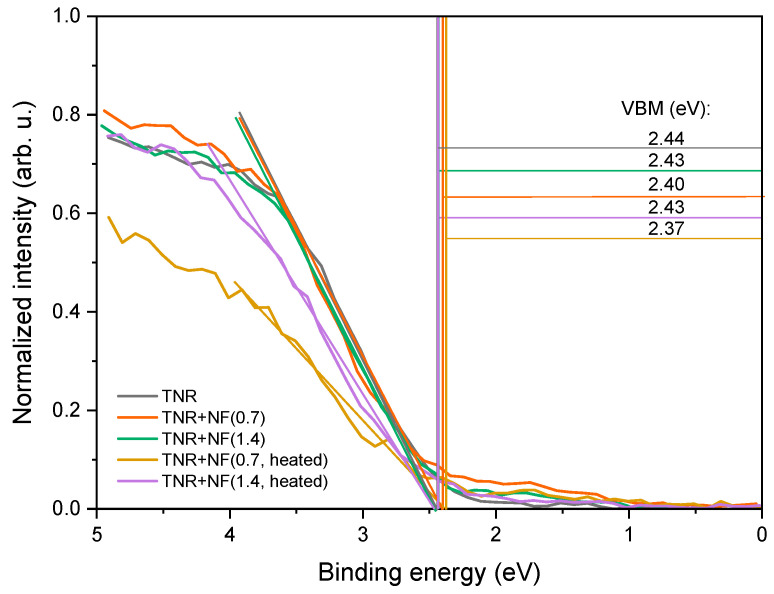
Determination of VBM for pure TNR support and Au/TiO_2_ catalysts by means of XPS analysis.

The Schottky barrier forms at the interface between the plasmonic metal and the semiconductor and monitors the passage of hot charge carriers at the interface, in our case, at the interface between gold and TiO_2_. The height of the Schottky barrier is calculated using the following equation:

(1) $$\emptyset_{SB}=\emptyset_{M}-\chi$$

where $\emptyset_{M}$ represents the work function of Au and χ represents the electron affinity of the TNR catalyst support. Figure 8 shows valence band maxima (VBM) curves, and the calculated SBH values are listed in Table 1. The value of the SBH in the investigated Au/TiO_2_ catalysts corresponds to the differences between the VBM of pure TNR and the VBM of the Au/TiO_2_ catalysts and is in the range of 0.01–0.07 eV, which is consistent with reports in the literature where the SBH values are well below 1.0 eV [28,70,71]. TNR + NF(0.7, heated) and TNR + NF(1.4, heated) samples exhibit higher SBH than samples before heating.

#### 2.2.3. UV-Vis DR, PL and EPR Analyses

**Figure 9:**
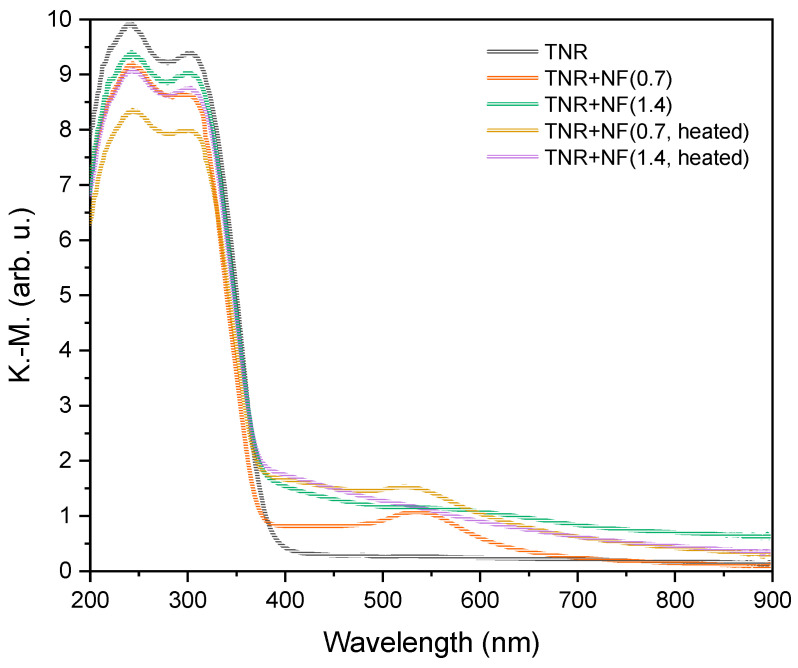
UV-Vis DR spectra of the TNR support and catalysts containing 1.0 wt.% of Au.

Figure 9 shows the results of the UV-Vis DR measurements. There is intense light absorption at wavelengths below 400 nm due to the presence of TiO_2_, and a hump in the visible range (up to 700 nm) caused by the deposited gold particles. The humps are not equally intense, which is due to the heterogeneous distribution and the differences in the shape of the Au ensembles. As expected, the most intense hump was observed in the TNR + NF(0.7) sample, which has well-defined Au NFs on the catalyst surface. The hump for the TNR + NF(1.4) sample is wider and of lower intensity, which is due to the heterogeneous distribution of the deformed Au NFs on the catalyst surface (Figure 2, Figures S3 and S4). In addition, the hump of the TNR + NF(0.7) sample is at 541 nm, which is lower than that of the TNR + NF(1.4) sample (576 nm). It is known that smaller Au NPs have the LSPR maxima at lower wavelengths [72]. It could thus be demonstrated that the Au NPs in the TNR + NF(0.7) sample (which has a perfectly flower-shaped structure) are smaller than those in the TNR + NF(1.4) solid (Table 1). In addition, the TNR + NF(1.4) sample exhibits a heterogeneous distribution and aggregation of Au particles, as shown in Figure S5a. This distribution is unfavorable as it limits the light absorption properties, which means that the TNR + NF(1.4) sample is likely to exhibit a limited photocatalytic response when exposed to visible light. The observed increase in LSPR for the TNR + NF(0.7) sample is also due to the presence of Au edges in the NFs, where the LSPR effect is most pronounced [28]. The UV-Vis DR spectra for heated Au/TiO_2_ samples differ from the spectra of the catalysts prepared at room temperature (Figure 9). The intensity of the humps is significantly lower for the heated samples as the plasmon effects are reduced due to the transformation of the Au NFs into spherical particles (Figure 2). Finally, the presence of plasmonic effects in the TNR + NF(0.7) catalyst upon illumination with visible light was demonstrated by a specially designed experiment described in the Supplementary Information.

**Figure 10:**
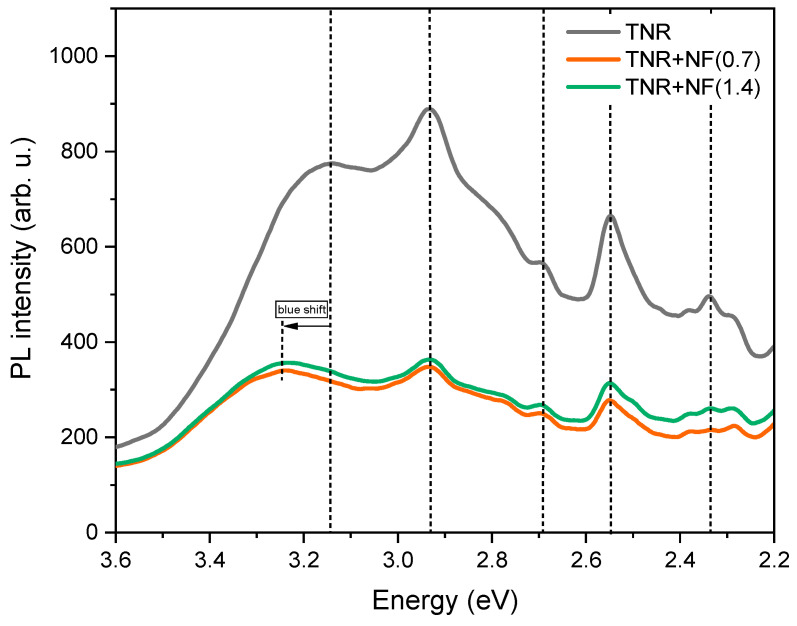
Solid-state photoluminescence (PL) spectra of the investigated materials.

Assuming that radiative relaxation plays a major role in the fate of charge carriers in semiconducting photocatalysts, compared to thermal and quantum relaxation, the photoluminescence technique represents an appropriate tool for the evaluation of their “lifetime”. A PL curve of a solid to be used for heterogeneous photocatalysis should be as low as possible (i.e., the recombination rate of the charge carriers would be low, resulting in low light emission). In this way, the charge carriers (i.e., holes and electrons) would be available for the redox processes occurring on the catalyst surface. The results of PL solid-state measurements of Au/TiO_2_ catalysts examined in the present study are shown in Figure 10. One can see that the TiO_2_ support exhibits a high charge carrier recombination rate, but it decreases with the addition of plasmonic metals (e.g., gold, regardless of the nanoparticle shape). TiO_2_ is present in the synthesized catalyst in the anatase form, as confirmed by the peak at 3.17 eV in the PL spectrum, which is characteristic of the anatase form of TiO_2_. In the Au/TiO_2_ samples, a peak shift from 3.17 to 3.25 eV can be seen compared to the TNR support, referred to as the blue shift. This range shift is due to the gold nanoflowers suppressing indirect phonon-assisted transitions in anatase TiO_2_. The peak at an energy of 2.9 eV indicates the lowest indirect transition, Γ_1b_ → X_1a_. The peaks in the PL spectra at energies of 2.7, 2.55 and 2.34 eV are characteristic of defects in the TNR support, oxygen vacancies and shallow trap levels. The results of PL measurements for the samples with the addition of gold particles in the form of nanoflowers show that adding gold increases the lifetime of the charge carriers. However, the curves of the Au/TiO_2_ samples are very similar, probably due to the similar size of the Au ensembles, which also explains the similar Schottky barrier (SB) formation.

**Figure 11:**
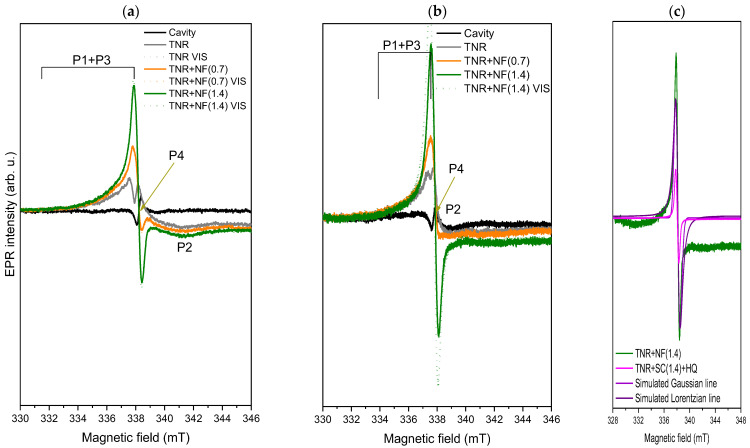
Solid-state EPR spectra of investigated samples under dark and visible-light illumination at (**a**) room temperature and (**b**) temperature of liquid nitrogen. (**c**) contains a simulation of the Lorentzian and Gaussian line shapes in comparison for the simulated impregnation material (TNR + Na-citrate(1.4) + HQ) measured at RT, and for the TNR + NF(1.4) sample examined at the temperature of liquid nitrogen.

To obtain further information about the properties of the analyzed samples, we carried out the solid-state EPR measurements in the dark and under illumination with visible light. Figure 11a shows the EPR spectra obtained at room temperature (RT), where clear differences between the samples can be recognized. For the pure TNR component, we can observe the peaks corresponding to Ti^3+^ (g_┴_ for P1 and g_‖_ for P2) and O-vacancies (P3) (see g-values in Table S2) [73,74]. This also agrees with the solid-state PL measurements (Figure 10), where we observe transition characteristics for O-vacancies. The presence of Ti^3+^ (at crystallization defects or at surface sites) and of O-vacancies could be due to the hydrothermal synthesis used to prepare the TNR material [75,76]. In addition, the broad, mixed signal P1 + P3 suggests that the Ti^3+^ is predominantly present at the surface of the material [77]. This can be advantageous as Ti^3+^ can absorb visible light [78]. For both Au-containing samples, we can observe changes in the EPR spectra. In the TNR + NF(0.7) sample, a new signal P4 can already be seen, from which shape we can ascribe it to electrons that are probably located at the C centers of organic compounds (g-value of 2.003), such as in g-C_3_N_4_ [79], or as observed by Caretti et al. [80] in their studies using Au/TiO_2_ and Na-citrate as reducing agents. This is probably due to the Na-citrate and HQ used in the synthesis of the materials. These organic compounds (P4 peak) overlap with the P1 + P3 peaks of pure TNR. However, the P2 peak is still present. The broad shape of the P4 peak indicates a disordered environment at the surface (heterogeneity), probably due to the presence of different types of species from TiO_2_, Au or carbon-containing species [81].

To gain further insight into the character of the organic compounds affecting the EPR spectra (P4), we performed the solid-state EPR measurements of pure Na-citrate and HQ at RT (Figure S9a). Surprisingly, both pure components are EPR-silent, indicating that Na-citrate and HQ likely reacted with the TiO_2_ to form EPR-visible organic compounds. Alternatively, the organic compounds formed between HAuCl_4_, HQ and Na-citrate during the synthesis could react with TiO_2_ to produce this EPR signal (Figure 11a, P4). This was also suggested by the decrease in PL signal between 2.6 and 2.2 eV, which is characteristic of defects, O-vacancies and shallow trap sites in TiO_2_. Since the reaction between Na-citrate, HQ and TiO_2_ is required to form defects, we can tentatively assume that this must occur. From the SEM-EDXS analysis (Table 2), there is also a decrease in oxygen content from 46.0 wt.% (TNR sample) to 41.5 wt.% (TNR + NF(0.7) sample), further supporting the interactions between the organic compounds and TiO_2_ and the formation of additional O-vacancies (lower PL signal and higher EPR signal). The higher content of Na-citrate in the TNR + NF(1.4) sample is reflected in the increase of the characteristic EPR signal for electrons at the C centers of organic compounds. In addition, the increase in Na-citrate content probably introduced more oxygen into the materials, as we observed an increase in the oxygen content (Table 2) to 48.8 wt.% for the TNR + NF(1.4) sample. As a consequence, this increased the content of different species on the surface (heterogeneity) [81], which enhanced the EPR signal. When illuminated with visible light, the photogenerated electrons can be trapped in these C centers, which is reflected in a slight increase in EPR intensity if the content of these C centers is high enough (Figure 11a). In contrast, for TNR + NF(0.7) sample, the content of these centers is too low to effectively trap the photo-generated electrons at room temperature. However, one must be careful because if Na-citrate and HQ react with the TiO_2_, the surface becomes contaminated with these compounds, and we lose the ability of the Ti^3+^ on the surface to harvest visible light. Additionally, the new compounds could be incorporated into the TiO_2_ or located between Au and TiO_2_ at the interface, negatively influencing the carrier mechanism. The latter could limit the photocatalytic activity of the TNR + NF(1.4) sample. When we compare the g-values in Table S2, we can see that the position of the peaks does not shift with the increase of Na-citrate, indicating that the same species are present in both Au-contaminating materials.

To obtain further information on the origin of the P4 signal, we also simulated the impregnation phase in ethanol (Figure S9b,c). To simulate the chloride anions, we used an appropriate amount of NaCl. Figure S9b shows that TNR with only Na-citrate (0.7) does not generate a P4 signal. Furthermore, visible-light illumination does not increase the present signals. In contrast, the TNR + HQ sample or the combination of all three components (TNR, Na-citrate and HQ) generates the characteristic P4 signal (Figure S9c). Since the signal has a too high intensity, we have quenched the detection parameters to obtain Figure S9d, from which we can recognize a smooth, Gaussian P4 (simulated in Figure 11c for the TNR+ Na-citrate(1.4) + HQ sample) signal with a g-value around 2.003. The Gaussian line shape indicates inhomogeneous broadening, probably due to the dipole-dipole interaction between the organic compounds and the different superpositions of many individual (Lorentzian) components [82,83]. A greater heterogeneity in the Au-containing samples has already been mentioned, which will now be explained. In Figure S9d, the TNR + HQ sample exhibits the highest signal, and the intensity decreases with the addition of Na-citrate(0.7). The additional increase in Na-citrate(1.4) reduced the signal even more. This probably means that the HQ in ethanol reduces some organic compounds that remain on the TNR surface after drying. The addition of Na-citrate acts like a competition reaction, so the signal decreases because either (a) silent EPR species are formed or (b) the organic compounds are not adsorbed on the TNR surface. As can be seen in Table 2, the C content in the investigated samples increases when we increase the content of Na-citrate, which means that the most plausible explanation is option (a). Furthermore, the signal decreases when both samples are heated to 300 °C, as the carbon species are likely partially removed from the sample. Most interesting is the effect of illumination with visible light. No sample in Figure S9d showed an increase in EPR after illumination (only shown for the TNR + HQ sample), indicating that the C compounds of the surface do not absorb visible light. However, as can be seen in Figure 11a,b (see explanation below), the increase in signal due to visible light is most pronounced when the C-compounds are present on the surface of synthesized Au-containing solids. This means that the C-containing compounds do not absorb visible light but can capture the electrons generated, which could reduce the materials’ photocatalytic efficiency. Another observation from Figure 11a is the lack of a peak for the hot electrons generated by Au or of signals correlating with Au. Since the Au nanoparticles are about 40 nm in size, the conduction band electrons of Au cannot be detected by EPR spectroscopy, which explains the absence of the signal [84].

To obtain a more complete picture of the properties and charge dynamics, we also performed the EPR solid-state measurements at the temperature of liquid nitrogen (LN2). The results illustrated in Figure 11b show similar shapes of the EPR spectra as in the RT measurements, with all P1–P4 signals present. When the measurements were performed at LN2, the accumulation of the generated electrons could be observed as the EPR signal increased for all samples under visible-light illumination. However, the strongest increase was again observed in the TNR + NF(1.4) sample with the highest proportion of Na-citrate (C centers from organic compounds). This could now explain the absence of the peak for the hot electrons of Au injected into TiO_2_. The hot electrons could be immediately captured by the organic compounds at the surface between the Au/TiO_2_ interface so that they cannot be detected. As mentioned above, this could limit the photocatalytic activity of the TNR + NF(1.4) sample since most of the electrons generated by visible light could be trapped by the organic compounds left over from the synthesis. The dominant effect of these organic C compounds can also be seen in the shape of the EPR spectra in Figure 11b. This is due to the fact that the P2 signal is present in the spectrum of the pure TNR, while it disappears in the spectra of the Au-containing samples. Moreover, the shape changes to a more Voigt line shape (a combination of Gaussian and Lorentzian line shape functions) with the increase in Na-citrate content [82,83]. As it can be seen in Figure 11c for the TNR + NF(1.4) sample, at a lower magnetic field, the shape resembles the Lorentzian line. However, at a higher magnetic field, the shape changes to a more Gaussian shape. As we observed for the simulated material (without Au, Figure 11c), the shape is pure Gaussian, probably due to the dipole-dipole interactions of the organic compounds on the surface of TNR. Now we have the additional presence of Au, which interacts with TiO_2_, thus partly disturbing the dipole-dipole interactions [80,82]. This again proves that the organic compounds on the surface influence the optoelectronic and transfer mechanisms of the Au/TiO_2_ interface (capture of photogenerated electrons, Figure 11b). In addition, the RT-EPR spectra of both samples after heating (Figure S10a) show the same shape as the LN2-EPR spectra (Figure 11b) with a much higher EPR intensity (the same g-value of 2.004 as for P4 in the unheated samples). The organic species were probably more exposed due to the rearrangement of the Au-NP and covered a larger area of the Au NPs. It is also possible that the signal increases due to the formation of paramagnetic centers from EPR-silent organic compounds on the surface of the materials due to the heat treatment. Surprisingly, the heated samples show no increase in intensity upon illumination with visible light (Figure S9c), indicating that no new photogenerated electrons were captured. When the C-containing components cover the Au NPs (spheres) and the majority of the TiO_2_ surface, they cannot absorb visible light and cannot form charge carriers. Thus, no electrons could be absorbed by the C-containing compounds, which explains the absence of an increase in the EPR signal (Figure S10b). This finding could indicate that the two heated samples and the TNR + NF(1.4) sample could exhibit poorer photocatalytic activity compared to the TNR + NF(0.7) catalyst.

### 2.3. H_2_-Assisted NO_2_ Photocatalytic Reduction

**Figure 12:**
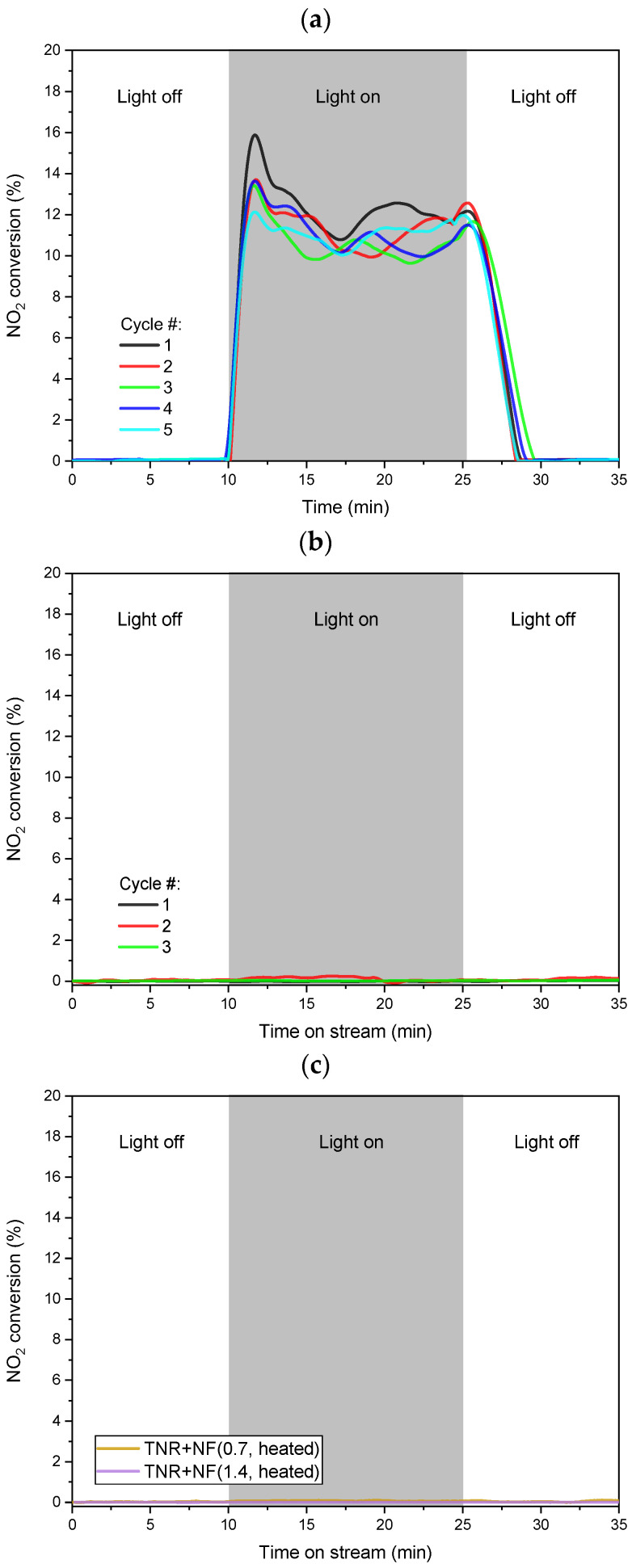
Visible-light assisted NO_2_ conversion obtained at 30 °C over (**a**) TNR + NF(0.7), (**b**) TNR + NF(1.4), (**c**) TNR + NF(0.7) and TNR + NF(1.4) samples heated at 300 °C.

The photocatalytic activity of the prepared Au/TiO_2_ catalysts was investigated by H_2_-assisted NO_2_ reduction by monitoring the decrease in NO_2_ concentration (as an ion current) in the photocatalytic flow reactor under visible-light illumination. The NO_2_ conversions shown in Figure 12 were calculated using the calibration curve generated at the end of each experimental run. The results of a blank experiment with an empty reactor (Figure S11a) confirm that the reactor walls contribute insignificantly to the disappearance of NO_2_ under visible-light illumination. The same was observed when only a bare TiO_2_ support was used (Figure S11b).

In the case of the TNR + NF(0.7) sample, there was a reduction in NO_2_ observed as soon as the light source was switched on (Figure 12a). The initial increase in NO_2_ conversion could be due to the formation of active species from –OH groups and surface-adsorbed water. However, over time, these are depleted, and we can observe a steady-state NO_2_ turnover after 15 min of visible-light exposure (Figure 12a) [85,86]. Furthermore, this spike becomes smaller with each cycle as fewer “fresh” –OH groups and water molecules are available on the surface of the sample. The activity of the TNR + NF(0.7) sample is expected as the Au NPs exhibit the LSPR effect (UV-Vis DR measurements), and the sample contains Ti^3+^ (XPS and EPR measurements), which can utilize visible light. In contrast, the TNR + NF(1.4) sample showed no ability to reduce NO_2_ under visible light (Figure 12b), which is quite surprising. None of the expected products were detected, which means that the inhibitory effect must occur in the early stages of the photocatalytic mechanism (adsorption of NO_2_, activation of NO_2_, etc.). To understand this phenomenon and provide possible explanations, we need to consider the results of UV-Vis DR, PL and EPR measurements. This could explain the non-activity of the TNR + NF(1.4) sample:

1. In the case of the TNR + NF(1.4) sample, the Au NPs have the form of deformed nanoflowers (TEM, Figure 2). The LSPR effect is most pronounced at the edges of the Au NPs (LSPR hot spots) and thus favors a nanostar or nanoflower-like shape of the Au NPs [87,88]. Since the form of Au NPs in the sample with the high Na-citrate content is more similar to a nanosphere, the LSPR effect is limited. This was also observed in the UV-Vis DR spectrum (Figure 9) due to the absence of the characteristic hump. Moreover, the Au NPs could partially behave like metals due to the low Schottky barrier (Table 1). Consequently, the utilization of visible light is limited.
2. As we found in the EPR measurements, the C-containing species left over from the synthesis probably cover most of the surface of the TNR + NF(1.4) material. This acts as a protective shield that limits the absorption of visible light and hinders the adsorption of NO_2_. NO_2_ is probably activated in a similar way to NO [89] by the transfer of electrons from the excited metal to the anti-bonding π orbital of NO_2_. As access to the NO_2_ molecule is restricted, it is not activated and cannot be further reduced to other products.
3. In addition, the C-containing species have a high tendency to trap (scavenge) photogenerated electrons (EPR measurements), which means that they intercept the charge carriers. If more Na-citrate is used in the synthesis, more C-containing species are likely to remain near or at the Au/TiO_2_ interface after the synthesis process. Therefore, in the case of the TNR + NF(1.4) sample, they can easily capture the photogenerated electrons of Ti^3+^, which means that they cannot be injected into the Au NPs even with a low Schottky barrier and vice versa. Even if the NO_2_ molecules are adsorbed or could interact with the Au NPs, the electron transfer cannot take place as no electrons reach the Au NPs. In the case of the TNR + NF(0.7) sample, some of the photogenerated electrons are transferred to the Au NPs, even with some content of the C-containing species (electron scavengers), where they can activate H_2_ or NO_2_. Similar results for analog systems were obtained by Huang et al. [90] and Siemer et al. [91]. This is possible for the TNR + NF(0.7) material as the Au NPs have sharper edges in the nanoflower structure and thus provide an improved Au/TiO_2_ interface as well as an enhanced LSPR effect at the hot spots (Au NP edges) [87,88].

In the case of the TNR + NF(0.7) sample, we observed fewer limiting factors and an improved LSPR effect compared to the TNR + NF(1.4) solid. This led to the observed conversion of NO_2_ at room temperature under only visible-light illumination. The reduction of NO_2_ with H_2_ produces three products: NO, N_2_O and N_2_. NO and N_2_O are important greenhouse gases and toxic, so N_2_ is the most desirable product due to its non-toxic properties. Figure S12 shows the MS ion current values for the three detectable main products as a function of time and illumination with visible light. The relative selectivity of the investigated catalysts was calculated by integrating the area under the ion current curves for each product [40] at the investigated visible-light illumination time. The results shown in Figure S12 (inset) indicate that the TNR + NF(0.7) catalyst exhibited a selectivity of 48.9% for N_2_ formation and 23.1 and 28.0% for NO and N_2_O, respectively. As observed, N_2_O was one of the by-products formed. Its formation could be due to the reaction between the generated NO molecule and O-vacancies, which is similar to the report by Diebold et al. [92] in the study with the rutile polymorph of TiO_2_. Furthermore, we can hypothesize that the N_2_O is generated by the lateral interactions of the catalyst surface with NO [93]. Due to the presence of activated hydrogen, the N_2_O (1+ oxidation state) can be further reduced to N_2_ (zero valent state). This is probably one of the reasons why the first reduction product, NO, has such low selectivity. Another reason is the further reduction of NO to N_2_ [94]. This is probably favored by the O-vacancies in TiO_2_, as observed by Wu and van de Krol [95]. According to our hypothesis, the NO_2_ is initially activated by the interactions between the antibonding orbitals in the NO_2_ and the electrons in the Au NPs. Together with the activated H, a reduction step probably takes place, and NO is formed. The NO is then probably desorbed from the vicinity of the gold and adsorbed on the O-vacancies in the TiO_2_ [96]. There, they form oxygen surface adsorbed O-N species. Due to the mobility at the O-vacancies, two molecules of these O-N species can meet at the surface and form N_2_(g) [95], as this is thermodynamically favorable and represents an exothermic reaction. Consequently, the heat generated contributes to the formation and release of O_2_, rendering the O-vacancy inactive (neutral). Since the holes created by the absorption of visible light by Ti^3+^ are present on the surface of TiO_2_, they can convert the neutral oxygen vacancies to the normal 2+ oxidation state [95], making them active again. Overall, the presence of both processes enables high selectivity in the generation of N_2_.

To test the longevity of the prepared TNR + NF(0.7) sample, we performed NO_2_ reduction under visible light in five consecutive runs using the same catalyst batch. After each NO_2_ reduction run, we purged the TNR + NF(0.7) catalyst for 60 min in a flow of 70 mL/min of pure Ar. Figure 12a shows that the TNR + NF(0.7) sample exhibits stable photocatalytic activity regardless of the number of catalytic runs.

The results in Figure 12c show that no NO_2_ reduction assisted by visible light was observed for the TNR+ NF(0.7, heated) and TNR + NF(1.4, heated) samples. The heating caused the Au NPs to form spherical particles, as they are the most thermodynamically stable form. This limits the LSPR effects and reduces the utilization of visible light. In addition, heating increased the height of the Schottky barrier, which limits the transfer of photogenerated electrons. However, a certain photocatalytic activity should be retained. The lack of NO_2_ conversion can again be explained by the C-containing species left over from the synthesis. As already mentioned, these species can capture and trap photogenerated electrons, which limits or even completely eliminates photocatalytic activity (TNR + NF(1.4) sample). When the samples are heated, the Au NPs undergo structural rearrangements. Since most of these C-containing species are probably located near the Au/TiO_2_ interface, they are also rearranged together with the Au NPs. They probably completely cover the Au NPs and the surroundings of the Au/TiO_2_ interface, so that the photogenerated electrons are trapped in both heated photocatalysts. This is also true for the previously active TNR + NF(0.7) sample. This similar trapping effect was already assumed in the EPR measurements in Figure S10b, as the signals for both heated photocatalysts have a similar intensity and shape. The assumptions from the EPR measurements are now again confirmed, as they are consistent with the lack of NO_2_ conversion in both samples.

Using Au nanoflowers deposited on TiO_2_, we have shown that it is possible to convert NO_2_ to N_2_ when illuminating the photocatalyst with visible light at room temperature. However, care should be taken in the choice of synthesis method (Na-citrate and HQ content, heating, etc.) as this can affect the charge carrier dynamics and render the photocatalyst completely inactive.

## 3. Experimental

### 3.1. Materials

Sodium hydroxide (NaOH, ≥98%, Merck, Darmstadt, Germany), hydrochloric acid (HCl, fuming 37%, ≤1 ppm free chlorine, Merck, Darmstadt, Germany), gold (III) chloride hydrate (HAuCl_4_·xH_2_O, ~50% Au basis, Sigma Aldrich, Taufkirchen, Germany), sodium citrate tribasic dihydrate, ≥99.0%, (HOC(COONa)(CH_2_COONa)_2_·2H_2_O, Sigma Aldrich, Taufkirchen, Germany), hydroquinone, ≥99.0%, (C_6_H_4_-1,4-(OH)_2_, Sigma Aldrich, Taufkirchen, Germany) and absolute ethanol (C_2_H_5_OH, ≥99.5%, Carlo Erba, Val de Reuil, France) were used as received. TiO_2_ precursor DT-51 was donated from the company CristalACTiV™, Thann, France.

All glassware used was previously cleaned with aqua regia and ultrapure water (with a resistivity of 18.2 MΩ cm). All aqueous solutions were prepared with ultrapure water and stored in the dark at 4 °C for a maximum of 24 h after preparation.

### 3.2. Catalyst Synthesis

The synthesis of titanium nanorods (TNR) was carried out by hydrothermal synthesis and is described in detail in our previous publications [32,97]. For the synthesis of Au NFs, gold seeds were first synthesized by the Turkevich method (1951) by citrate reduction [98]. The procedure consists of heating 100 mL of ultrapure water and 2.9 mL of gold solution (10 mM HAuCl_4_) to boiling and then adding 3.0 mL of a 1.0 wt.% Na-citrate solution. After the color changed to red, the gold seed suspension was stirred for an additional 10 min and then stored at 4 °C.

The gold seed suspension (the size of gold particles in the gold seed solution ranged from 12 to 16 nm (Figure S13a)) was then used to synthesize Au nanoflowers (NFs). Au NFs were synthesized by the hydroquinone reduction method, also known as the gold seed-mediated growth method [23]. In 100 mL of ultrapure water, 1.445 mL of 10 mM HAuCl_4_ solution, 2 mL of gold seed, 0.7 or 1.4 mL of 1.0 wt.% Na-citrate solution and 0.7 mL of 30 mM hydroquinone solution were added successively every 45 s. The synthesis of Au NFs was carried out at room temperature. After adding hydroquinone, the suspension immediately turned blue and was stirred for an additional 3 min. All solutions for the preparation of the nanoflowers were prepared no more than 24 h before synthesis. The prepared solutions were designated NF(0.7) and NF(1.4), with NF(0.7) containing a lower amount of Na-citrate (0.7 mL) and NF(1.4) containing a higher amount of Na-citrate (1.4 mL).

The deposition of Au NFs on the TNR support was performed by wet impregnation, using a similar procedure as in our previous publication [71], only the amount of solvent and stirring time were different. Then, 0.3 g of TNR and 100 mL of ethanol were dispersed in an ultrasonic bath for 10 min. To the mixture of ethanol and TNR, the entire amount of nanoflower suspension was added, and everything was mixed for 15 min. The resulting materials were then treated in a rotary evaporator at a bath temperature of 45 °C and further dried at room temperature. The synthesized catalysts were referred to as TNR + NF(0.7), where NF(0.7) was applied to TNR, and as TNR + NF(1.4), where NF(1.4) was applied to TNR. Both Au/TiO_2_ catalysts were heated in the photocatalytic flow reactor for NO_2_ reduction. The temperature was increased from 30 to 300 °C using a heating ramp of 10 °C/min and maintained at 300 °C for 30 min. We then cooled the treated materials, labeled TNR + NF(0.7, heated) and TNR + NF(1.4, heated), to 30 °C and rinsed them in a stream of pure Ar (70 mL/min) for 60 min.

The photocatalytic activity of the prepared Au/TiO_2_ catalysts was investigated by H_2_-assisted NO_2_ reduction by monitoring the decrease in NO_2_ concentration (as ion current) in the photocatalytic flow reactor under visible-light illumination.

### 3.3. Catalyst Characterization

Au/TiO2 catalysts were investigated using structural (TEM, SEM, XRD, N2 physisorp-tion), chemical (FTIR, XPS) and optoelectronic characterization (UV-Vis DR, PL, EPR). A transmission electron microscope (TEM, JEM-2100, JEOL Inc., Tokyo, Japan) and a scan-ning electron microscope (SEM, SUPRA 35 VP, Carl Zeiss, Oberkochen, Germany) equipped with an energy dispersive X-ray spectrometer (SEM-EDXS, Inca 400, Oxford In-struments plc, Abringdon, UK) were used to analyze the morphology and composition of the materials studied. For the TEM analysis, the NF suspensions were attached directly to the carbon support grids, while the solid samples were diluted in absolute ethanol, soni-cated for a few seconds for dispersion and then dripped to the carbon grids. For the SEM-EDXS measurements, only powdered samples, which were attached to an aluminum holder with double-sided carbon tape, were used. SEM-EDXS analysis of the investigated solids was performed at 10 randomly selected locations at a beam energy of 15 kV in area scan mode.

The crystallinity and phase composition of the synthesized catalysts were determined using an X-ray diffractometer (XRD, X’Pert Pro, PANalytical, Almero, The Netherlands) with Cu Kα1 radiation (λ = 0.15406 nm). Catalysts were scanned in the 2θ range between 5 and 90°, with step sizes of 0.033° and a 100 s step time.

N2 physisorption measurements were performed at −196 °C using a TriStar II 3020 analyzer from Micromeritics, Norcross, GA, USA. Before measurements, catalysts were placed in a freeze dryer for 24 h to degas the samples. The specific surface area was calcu-lated using the Brunauer-Emmett-Teller method (BET), while the Barrett-Joyner-Halenda method (BJH) was used to calculate the total pore volume and pore size distribution from the desorption branch of the acquired isotherms.

A FTIR spectrometer (Perkin Elmer, model Frontier, Waltham, MA, USA) was used for the ATR-FTIR measurements. For all spectra, an average of 32 scans was used in a range between 4000 and 400 cm^−1^ with a spectral resolution of 4 cm^−1^.

The CO chemisorption on the synthesized Au/TiO_2_ catalysts was measured by FTIR-DRIFTS analysis (Perkin Elmer, model Frontier). Then, 10 mg of the catalyst sample was weighed into the FTIR-DRIFTS cell (Pike Scientific, Madison, WI, USA), and the entire cell was purged with pure nitrogen and then subjected to a vacuum treatment (Pfeiffer Vacu-um, model HiCUBE, Aßlar, Germany) to clean the surface of the sample. The vacuum reached a value of 0.1 Pa in the cell. After vacuuming, the sample was exposed to a 1% CO/Ar stream and then to N2 again to analyze the possible chemisorption of CO on the catalyst surface.

Utilizing the Supra + device (Kratos, Manchester, UK) equipped with a monochroma-tor and an Al Kα excitation source, an X-ray photoelectron spectroscopy (XPS) investiga-tion was conducted. The studies were carried out using an analyzed spot size of 300 by 700 µm, and the powder samples were placed on double-sided carbon tape attached to the Si wafer. Charge neutralization was enabled during the analysis. Survey and high-resolution spectra were measured at 20 and 160 eV pass energies, respectively. The take-off angle of all the XPS measurements was 90°. ESCApe 1.5 software was utilized for both spectrum acquisition and data processing (Kratos). The intensity of the peaks was obtained using Shirley background correction.

UV-Vis diffuse reflectance (UV-Vis DR) spectra were measured for the catalysts stud-ied in the wavelength range between 200 and 900 nm at room temperature and a scan rate of 266.75 nm per minute using a UV-Vis spectrophotometer (Perkin Elmer, model Lambda 650, New York, NY, USA) equipped with a Praying Mantis DRP-SAP accessory from Harrick. For more accurate measurements between 400 and 800 nm, the scan rate was set to 100.61 nm per minute. All measurements were repeated five times to obtain accurate results. UV-Vis absorption spectra (Perkin Elmer, model Lambda 465, Shelton, FL, USA) for liq-uid-phase samples were measured at ambient temperature using a quartz cuvette with an optical path length of 10 in the wavelength range between 300 and 900 nm.

Solid-state measurements of the synthesized catalysts by photoluminescence (PL) were performed using a Perkin Elmer UV-Vis fluorescence spectrometer (model LS 55, Waltham, MA, USA). The scan speed was set at 200 nm per minute, the excitation wavelength was 300 nm, the excitation slit was 5 nm and the emission slit was 7.5 nm. The wavelength range was set from 300 to 600 nm.

An Adani CMS8400 EPR spectrometer (Minsk, Belarus) was used to obtain solid-state paramagnetic resonance spectra (X-band) at room temperature or at liquid nitrogen tem-perature. Mg(II)/MgO powder was used as a standard to determine the uncertainty of the measurements of ±0.0005. A powder sample was placed in a quartz sample tube and in-serted into the EPR spectrometer (9.4 MHz microwave frequency). The measurements at room temperature were carried out at 338.00 mT (sweep width 20 mT) with a modified amplitude of 450 µT and a power attenuation of 18 dB (gain value of 1 × 103). For one spectrum, 120 s with three consecutive measurements were used to obtain an average val-ue. The measurements at liquid nitrogen temperature were performed at 338.00 mT (sweep width of 20.00 mT) with a modified amplitude of 450 µT and a power attenuation of 18 dB with a gain value of 8 × 102. 120 s were used to obtain a spectrum with three consecutive measurements, which were averaged. In all cases, the sample was illuminated with visible light (Schott, model KL 2500 LED, the energy spectrum is shown in Figure S14) through the side channel of the EPR spectrometer for 10 min prior to measurement.

### 3.4. H_2_-Assisted NO_2_ Photocatalytic Reduction

Photocatalytic H_2_-assisted NO_2_ degradation reactions were performed in a temperature-controlled Praying Mantis reaction chamber from Harrick (model HVC-MRA-5) designed for Raman spectroscopy measurements. Catalysts were packed as thinly as possible (4 mm, 20 mg) to ensure the maximum absorption of light. A Pfeiffer Vacuum Omnistar mass spectrometer was connected to the outlet of the reaction chamber to allow real-time quantitative analysis of the gaseous products. Samples were pretreated in a flow of 70 mL/min of pure Ar for 180 min in the dark at room temperature. The reactor was then exposed to the reaction gas mixture of 5000 ppm NO_2_ and 5% H_2_ in Ar at a gas flow of 70 mL/min for 1 h to condition and stabilize the mass spectrometer. Then, a Schott KL 2500 LED light source (400 < λ < 700 nm, Figure S14), equipped with an optical fiber and light focusing lenses (Thorlabs Inc.) that precisely focused the light onto the catalyst bed (4.5 mm), was used for catalyst illumination.

## 4. Conclusions

This study systematically investigated the synthesis and characterization of nanoflower-shaped TiO_2_-supported Au catalysts, focusing on their structural, optical and electronic properties. The average size of Au particles was 32 nm for NF(0.7) and 35 nm for NF(1.4) suspensions. Wet impregnation of TiO_2_ nanorods with Au nanoflowers did not significantly change their size, indicating stability. TEM images and SAED patterns showed a uniform distribution of nanoflowers in the TNR + NF(0.7) sample and heterogeneous distribution in the TNR + NF(1.4) solid, which was also confirmed by SEM-EDXS and UV-Vis DR measurements. When heated to 300 °C, the nanoflowers turned into spherical Au particles as the centers of the nanoflowers melted. XPS analysis revealed metallic Au nanoflowers and shifts in the Au peaks, indicating oxygen vacancies and electron transfer from TiO_2_ to Au at the heterogeneous interface. Solid-state EPR measurements revealed Ti^3+^, oxygen vacancies and electron interactions with organic compounds on the catalyst surface. The TNR + NF(0.7) sample exhibited high photocatalytic activity for H_2_-assisted NO_2_ reduction under visible light. This activity was expected since the Au nanoparticles in the form of nanoflowers exhibit the LSPR effect at the edges. The stability of the TNR + NF(0.7) sample was demonstrated over several cycles, with heating affecting the morphology of the gold particles and the photocatalytic activity. Surprisingly, the TNR + NF(1.4) catalyst showed no ability to reduce NO_2_ under visible light, which can be attributed to deformed nanoflowers and carbon species hindering the utilization of visible light. Heating both samples reduces their photocatalytic activity by altering nanoparticle structures. In summary, the comprehensive characterization of TiO_2_-supported Au catalysts in the form of nanoflowers provides valuable insights into the effects of gold deposition on TiO_2_ nanorods, the formation of nanoflowers and the interaction with organic compounds on the surface, which contribute to a better understanding of catalyst behavior and form the basis for further investigations and potential applications in photocatalysis.

## Figures and Tables

**Table 1:** Results of TEM particle size measurements (Feret diameter) and XPS (Schottky barrier height (SBH)) analysis for solid Au/TNR samples.

Sample	TNR + NF(0.7)	TNR + NF(1.4)	TNR + NF(0.7, heated)	TNR + NF(1.4, heated)
Feret diameter of Au particles (nm)	30 ± 4	36 ± 4	35 ± 4	40 ± 4
SBH (eV)	0.04	0.01	0.07	0.01

Note: The average Feret diameter of Au particles in the solutions of the nanoflowers was 32 nm (sample NF(0.7)) and 35 nm (sample NF(1.4)). At least 80 Au particles were measured for each sample from TEM micrographs.

**Table 2:** Results of ^a^ SEM-EDXS, ^b^ ICP-OES and C-content analyses of the investigated TNR support and TNR + NF catalysts.

Sample		TNR	TNR + NF(0.7)	TNR + NF(1.4)	TNR + NF(0.7, Heated)	TNR + NF(1.4, Heated)
Ti	wt.%	54 ± 0.3	57.7 ± 0.3	50.4 ± 0.3	56 ± 0.3	57.1 ± 0.3
O		46 ± 0.3	41.5 ± 0.3	48.8 ± 0.3	43.1 ± 0.3	42.1 ± 0.3
Au		-	0.9 ± 0.2 ^b^ (0.98)	0.8 ± 0.2 (0.93)	0.9 ± 0.2 (1.03)	0.7 ± 0.1 (0.95)
C		0.3	1.2	1.7	1.0	1.4

^a^ Analysis conditions: voltage 15 keV, ZAF correction method. The instrument was calibrated with an analytical mono-block from MAC.

**Table 3:** Results of N_2_ physisorption (specific surface area (*S*_BET_), pore volume (*V*_pore_) and pore diameter (*d*_pore_)) and XRD (the apparent crystallite size of anatase was calculated from the diffraction peaks at 25°, using the Scherrer equation) analyses.

Sample	TNR	TNR + NF(0.7)	TNR + NF(1.4)
^a^ Apparent anatase crystallite size (nm)	17	17	17
*S*_BET_ (m^2^/g)	106	86	85
*V*_pore_ (cm^3^/g)	0.48	0.43	0.42
*d*_pore_ (nm)	18.3	19.9	19.9

^a^ The apparent values were calculated because the TNR support is non-spherical.

## Data Availability

The raw data supporting the conclusions of this article will be made available by the authors on request.

## Supplementary Materials

The following supporting information can be downloaded at: https://www.mdpi.com/article/10.3390/molecules29143333/s1, Figure S1: SEM images of the analyzed samples: (a) TNR, (b) TNR + NF(0.7), (c) TNR + NF(1.4), (d) TNR + NF(0.7, heated) and (e) TNR + NF(1.4, heated); Figure S2: Photographs of the synthesized catalysts: (a) TNR + NF(0.7), (b) TNR + NF(1.4), (c) TNR + NF(0.7, heated), and (d) TNR + NF(1.4, heated); Figure S3: TEM micrograph of Au NPs and corresponding experimental SAED pattern, which is rotational averaged (rot. avr.; in inset) and compared to ab-initio simulated SAED pattern for Au. Samples: (a) NF(0.7) and (b) NF(1.4); Figure S4: During evaporation on the rotary evaporator, the sample TNR + NF(1.4) became darker in the top layer and lighter in the bottom layer of the round bottom evaporation flask; Figure S5: (a) TEM micrograph of sample TNR + NF(1.4) showing anatase TiO_2_ nanorods (TNR) and Au accumulations, and (b) UV-Vis DR spectra for the top and bottom layer formed during synthesis of sample TNR + NF(1.4) in the evaporation flask; Figure S6: (a) ATR-FTIR spectra of used organic chemicals (Na-citrate, HQ), and (b) ATR-FTIR spectra of the TNR support with adsorbed Na-citrate and/or HQ; CO-DRIFTS analysis; Figure S7: The results of CO chemisorption measured with DRIFTS analysis. The orange curves show the DRIFTS spectra for the TNR + NF(0.7) sample, after 10 min of purging with 1% CO/Ar and after 5, 10 and 15 min of subsequent purging with N_2_. The green curves show the results for the TNR + NF(1.4) sample using the same protocol; Figure S8: (a) High-resolution C 1s spectra for TNR, TNR + NF(0.7), TNR + NF(1.4), TNR + NF(0.7, heated), and TNR + NF(1.4, heated) samples and (b) fitted Au 4f spectra; Figure S9: Solid-state EPR spectra obtained at RT for the synthesis components: pure Na-citrate (SC), hydroquinone (HQ) and a physical mixture of TNR and HQ (a). (b,c) contain EPR spectra measured at RT for samples of synthesis components prepared according to the synthesis procedure, except that an equivalent amount of NaCl was used instead of the gold precursor. (d) contains the results of the measurements with adjusted instrument parameters (gain factor of 5 × 10^1^) for the samples in (c). In all cases, visible-light (VIS) irradiation was accumulated for 10 min before the EPR spectra were recorded. ΔT in the sample designation means that the sample was heated at 300 °C in an inert atmosphere prior to the measurements; Figure S10: (a) Results of EPR analysis of heated and unheated catalyst samples measured in darkness and visible light. The gain value for (b) was 6 × 10^2^ for all samples to obtain the intensity of the EPR spectra within the detector limits. In all cases, visible-light (VIS) irradiation was accumulated for 10 min before recording the EPR spectra. ΔT in the sample designation means that the sample was heated at 300 °C in an inert atmosphere prior to the measurements; Figure S11: NO_2_, N_2_, NO and N_2_O mass spectrometer measurements for (a) the empty reactor and (b) the bare TNR. The reaction took place in a mixture of 5000 ppm NO_2_ and 5% H_2_ in Ar with a gas flow of 70 mL/min. The grey area shows the time during which the illumination with visible light was switched on; Figure S12: Time and visible-light illumination dependent NO_2_, NO, N_2_O and N_2_ MS ion current readings for the TNR + NF(0.7) sample to calculate the relative percent selectivity of the three main products (NO, N_2_O and N_2_) during the visible-light illumination interval (grey area). The results of the calculation are shown in the inset. The reaction was carried out in a mixture of 5000 ppm NO_2_ and 5% H_2_ in Ar carrier gas at a flow rate of 70 mL/min and 30 °C. The catalyst was exposed to the reaction mixture for 1 h before being irradiated with visible light; Figure S13: The results of the size distribution of (a) Au-seeds and Au NFs in suspension; (b) NF(0.7) and (c) NF(1.4) (80–100 measured Au NPs with TEM analysis); Figure S14: The energy spectrum of the Schott KL 2500 LED light source used in the process of H_2_-assisted NO_2_ photocatalytic reduction; Presence of plasmonic effects in TNR + NF(0.7) catalyst under visible-light illumination; Table S1: Results of SEM-EDXS analysis of the investigated TNR + NF(1.4) catalyst; Table S2: Calculated g-values from solid-state EPR spectra of investigated photocatalysts for P1 + P3, P2 and P4 signals. Samples marked with LN2 were measured at the temperature of liquid nitrogen. References cited in supplementary material [31,71,99,100].
