# Supplementary material for: Novel TiO2-Supported Gold Nanoflowers for Efficient Photocatalytic NOx Abatement
Source: Molecules. 2024 Jul 16;29(14):3333. doi: 10.3390/molecules29143333 (PMC11279453; doi:10.3390/molecules29143333)
Supplement: Supplementary file 1 [file molecules-29-03333-s001.zip › molecules-3053428-supplementary.pdf]

Supplementary Information

Novel TiO_2 supported gold nanoflowers for efficient photocatalytic NO_x abatement

Špela Slapničar[a], Gregor Žerjav[a], Janez Zavašnik[b], Matevž Roškarič[a], Matjaž Finšgar[c], Albin Pintar[a,*]

[a]Department of Inorganic Chemistry and Technology, National Institute of Chemistry, Hajdrihova ulica 19, SI-1001 Ljubljana, Slovenia

[b]Gaseous Electronics, Jožef Stefan Institute, Jamova cesta 39, SI-1000 Ljubljana, Slovenia

[c]University of Maribor, Faculty of Chemistry and Chemical Engineering, Smetanova ulica 17, SI-2000 Maribor, Slovenia

[*]Corresponding author. Tel.: +386 1 47 60 237. *E-mail address:* albin.pintar@ki.si (A. Pintar).

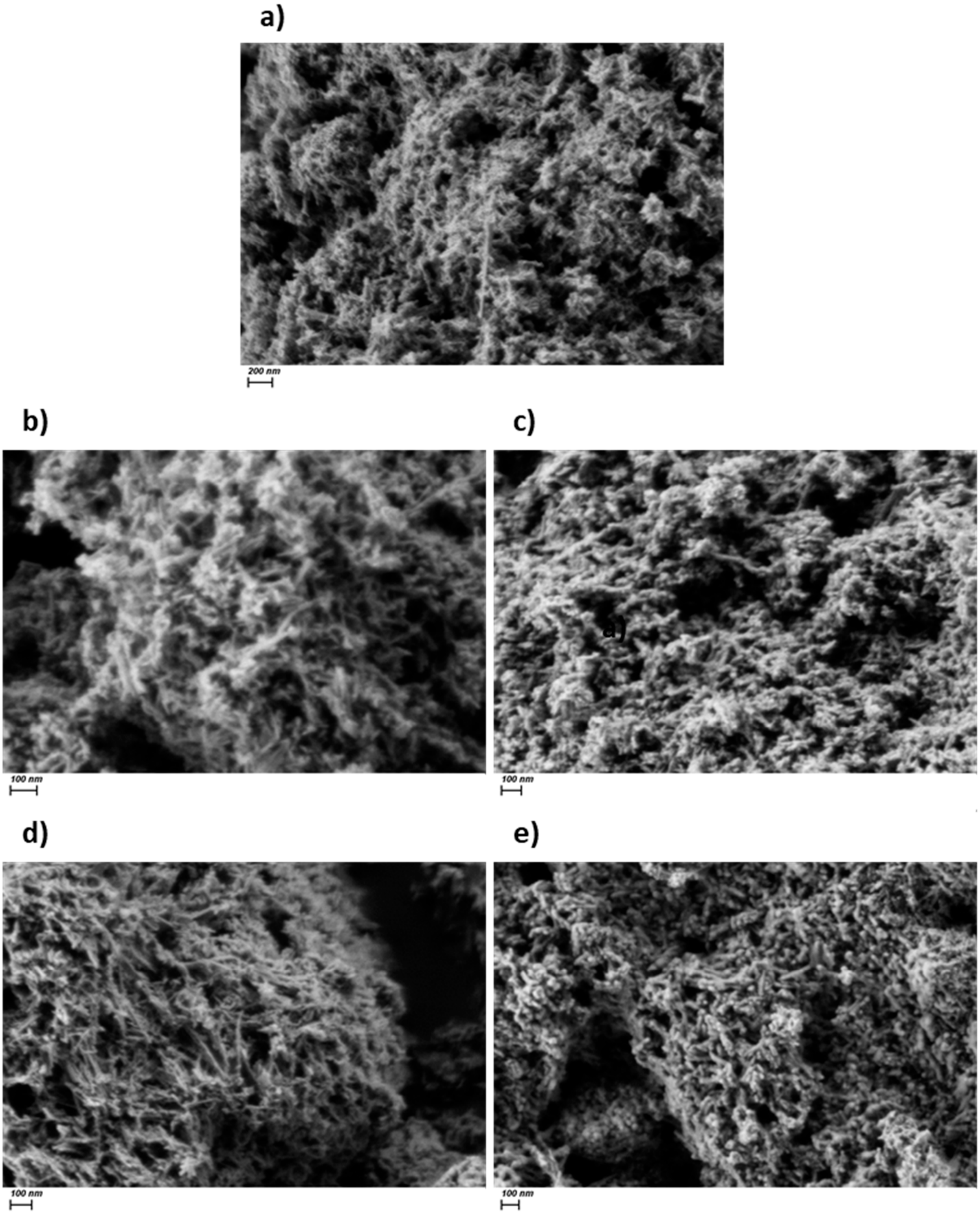

Fig. S1. SEM images of the analyzed samples: a) TNR, b) TNR+NF(0.7), c) TNR+NF(1.4), d) TNR+NF(0.7, heated) and e) TNR+NF(1.4, heated).

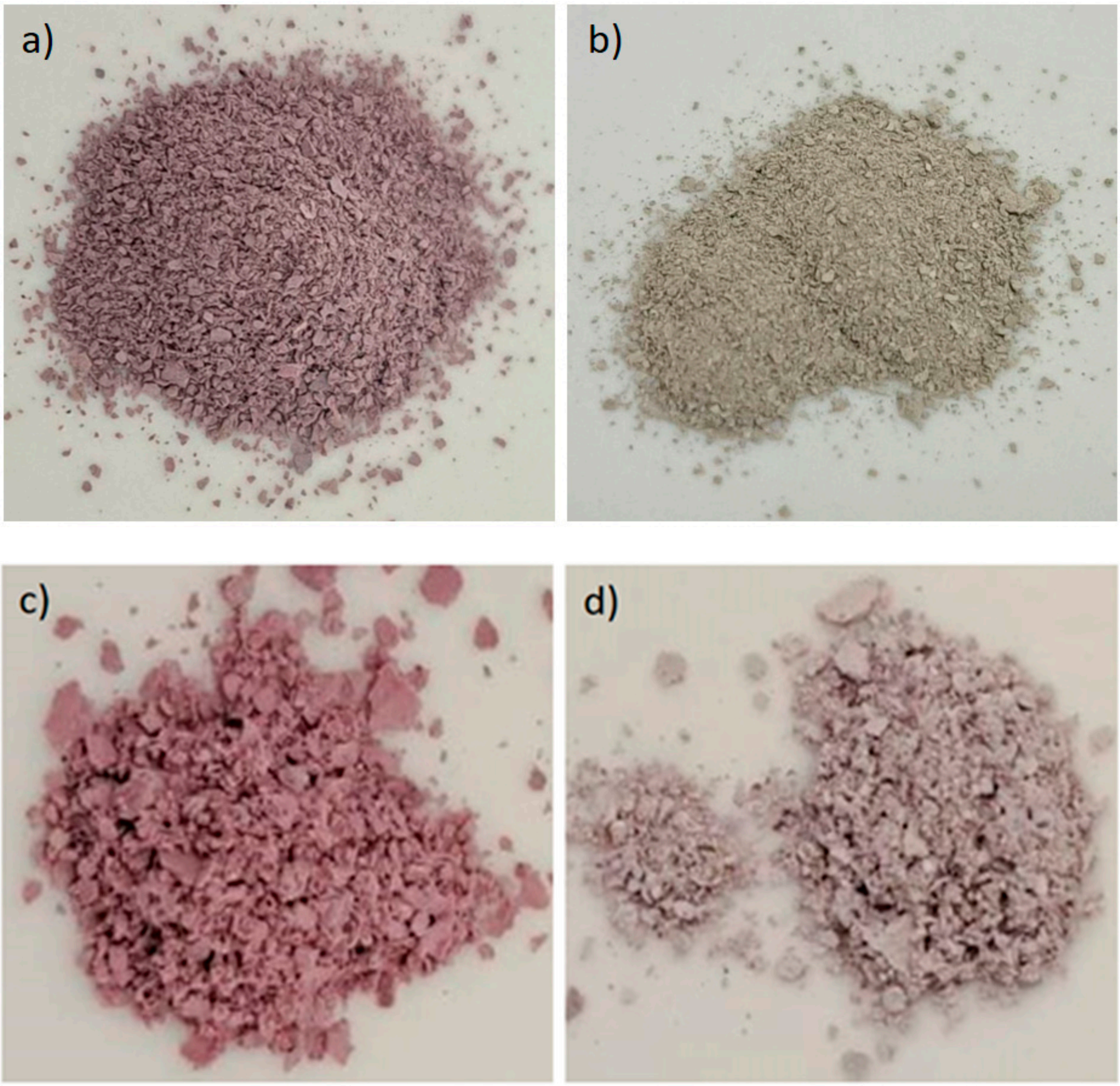

Fig. S2. Photographs of the synthesized catalysts: a) TNR+NF(0.7), b) TNR+NF(1.4), c) TNR+NF(0.7, heated), and d) TNR+NF(1.4, heated).

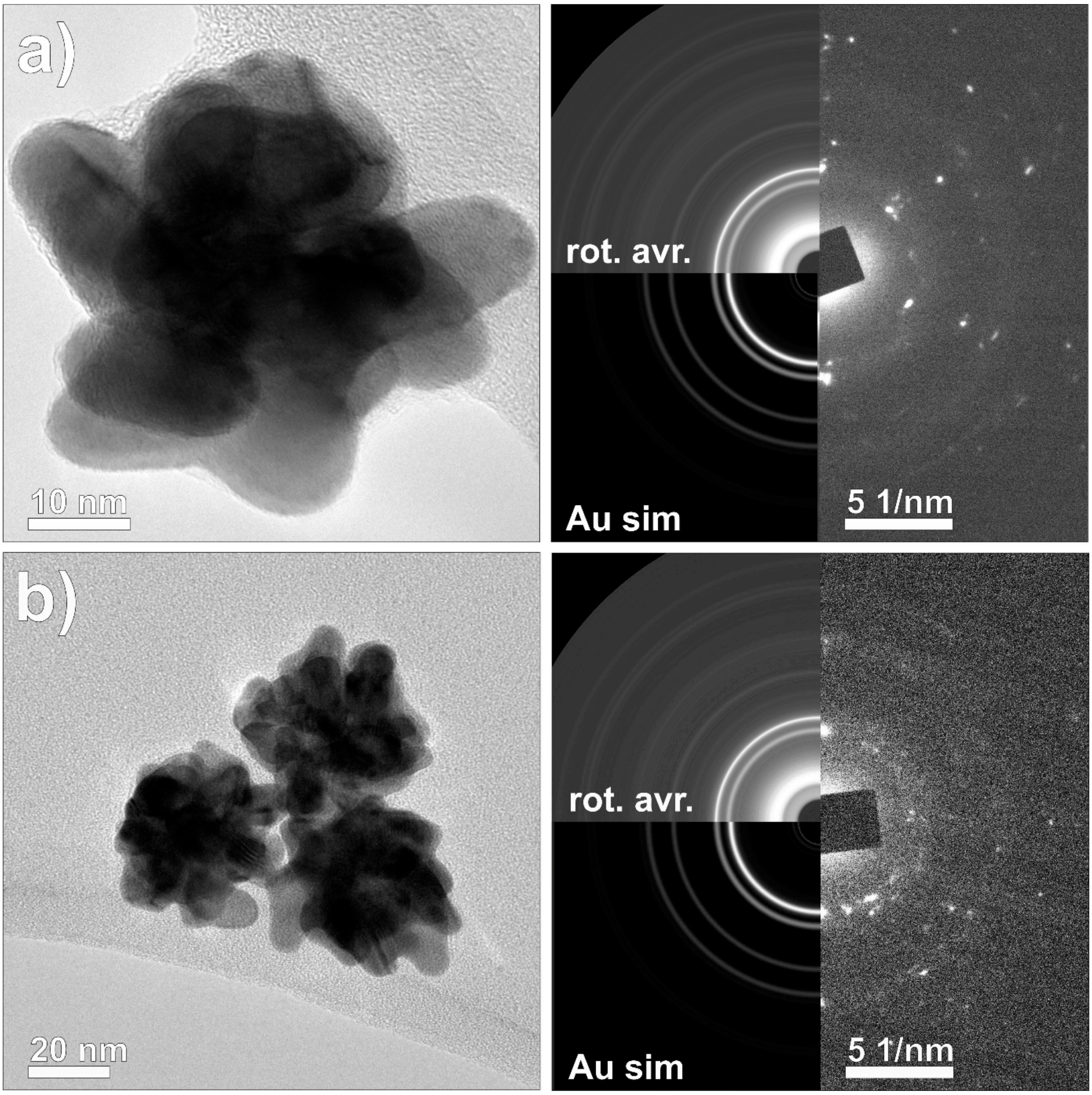

Fig. S3. TEM micrograph of Au NPs and corresponding experimental SAED pattern, which is rotational averaged (rot. avr.; in inset) and compared to *ab-initio* simulated SAED pattern for Au. Samples: a) NF(0.7) and b) NF(1.4).

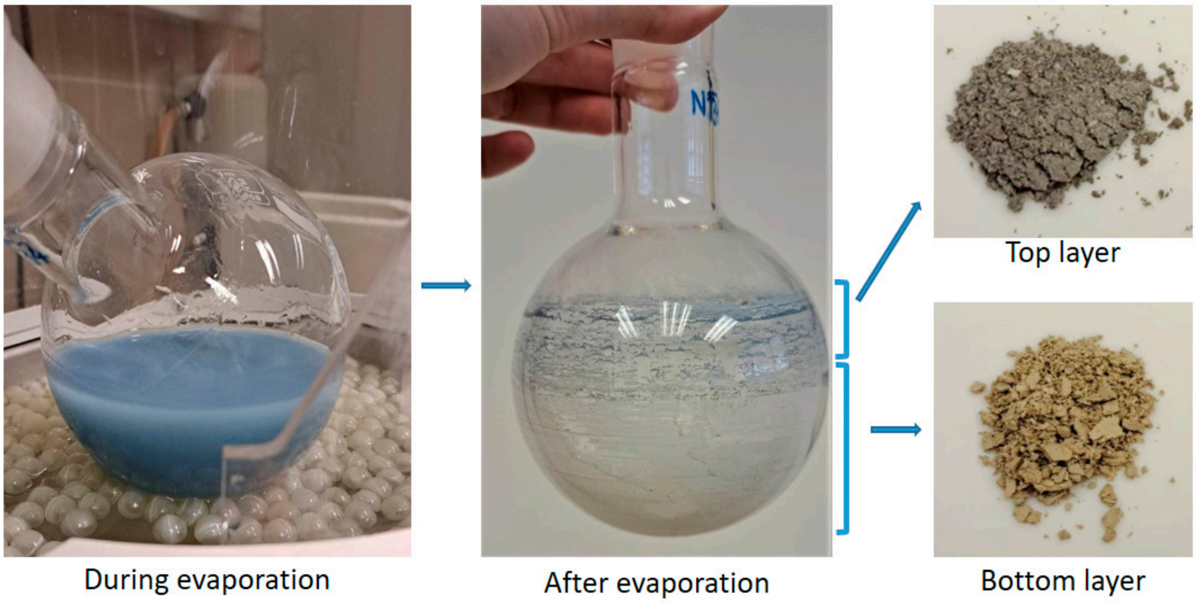

Fig. S4. During evaporation on the rotary evaporator, the sample TNR+NF(1.4) became darker in the top layer and lighter in the bottom layer of the round bottom evaporation flask.

a)

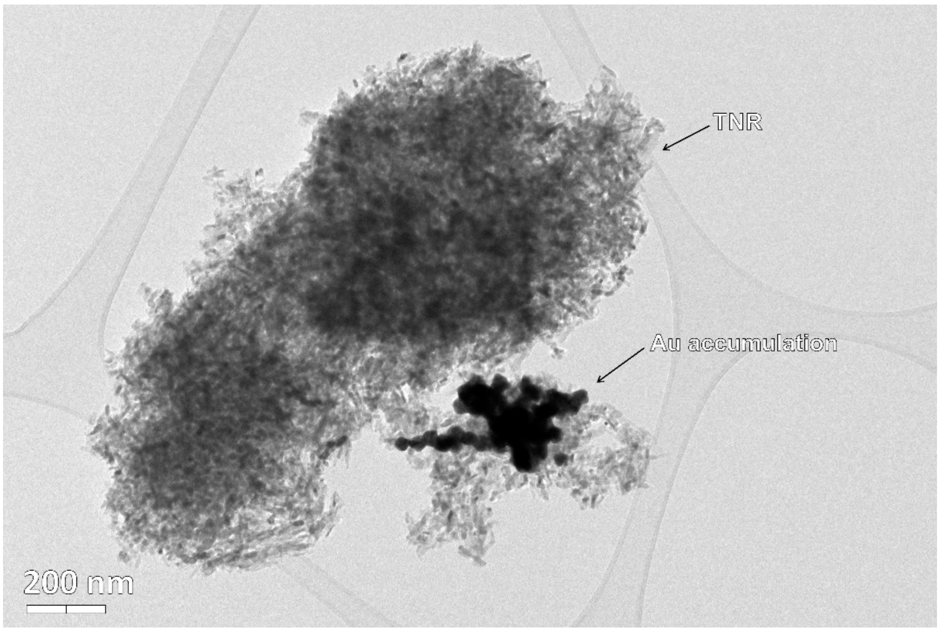

b)

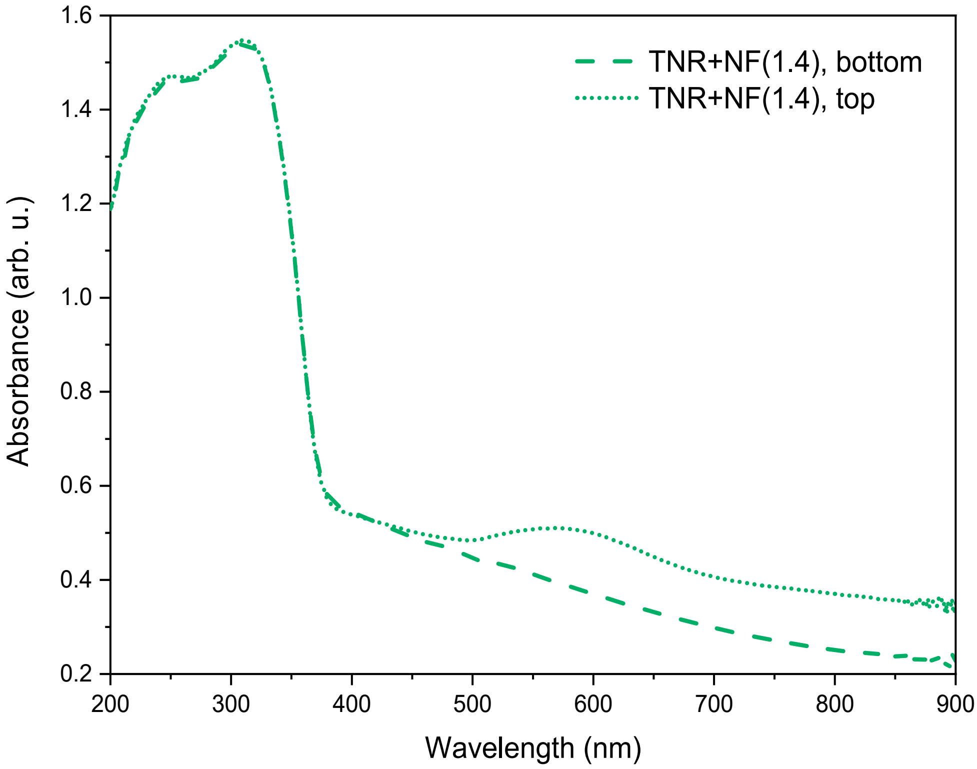

Fig. S5. a) TEM micrograph of sample TNR+NF(1.4) showing anatase TiO_2 nanorods (TNR) and Au accumulations, and b) UV-Vis DR spectra for the top and bottom layer formed during synthesis of sample TNR+NF(1.4) in the evaporation flask.

a)

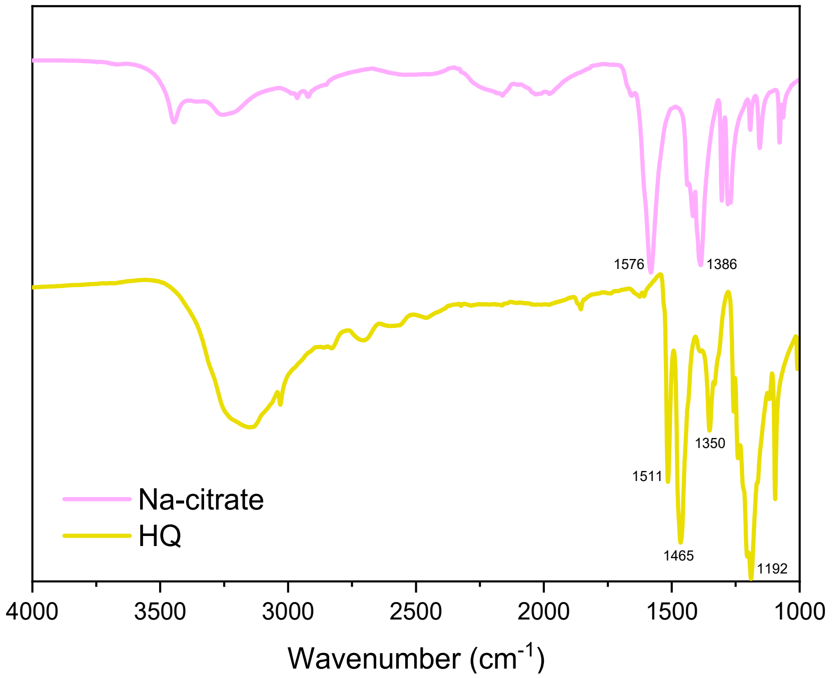

b)

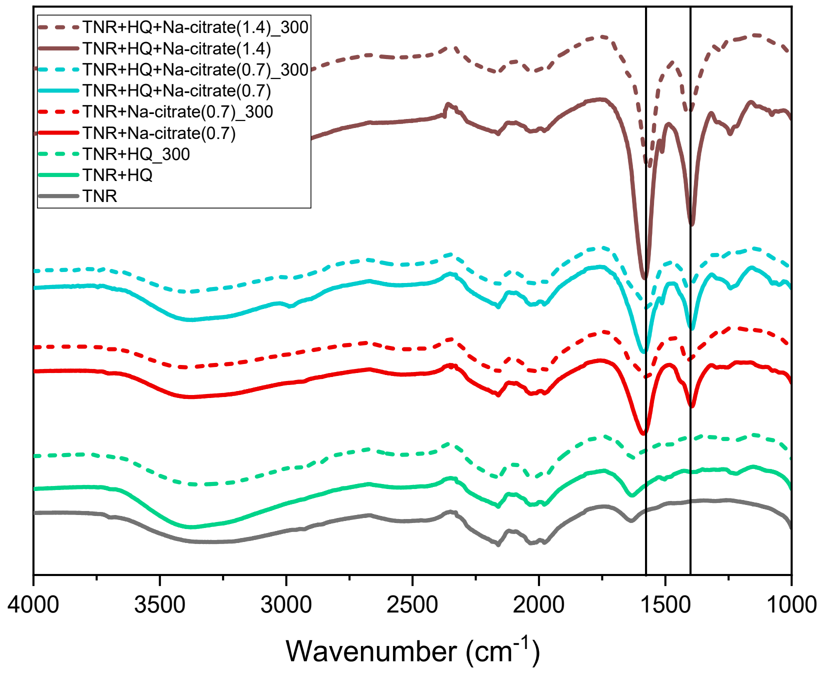

Fig. S6. a) ATR-FTIR spectra of used organic chemicals (Na-citrate, HQ), and b) ATR-FTIR spectra of the TNR support with adsorbed Na-citrate and/or HQ.

CO-DRIFTS analysis

The results of CO chemisorption measured by DRIFTS analysis are shown in Fig. S7. The first spectrum for each catalyst studied (TNR+NF(0.7) and TNR+NF(1.4)) was recorded immediately after rinsing the samples with 1% CO/Ar for 10 min. The CO peaks at wavenumbers 2100 and 2180 cm^{-1} are visible because the DRIFT cell was filled with CO. After completion of purging with 1% CO/Ar, purging with N_2 was initiated. After 5 minutes of purging with N_2 (and more), DRIFTS spectra were recorded in which the CO peaks were no longer present, indicating that CO was previously present only in the atmosphere of the DRIFTS cell but was not chemisorbed on the surface of the gold nanoparticles. The results are in agreement with the study of Boccuzzi et al. [1], who reported that chemisorption of CO on gold-coated catalysts is possible only for very small gold nanoparticles (below 10 nm). It should be noted that CO can also be adsorbed on TiO_2, which is usually indicated by the appearance of a peak at a wavenumber of 2050 cm^{-1}. However, this is only detected at very low temperatures (below -20 °C) and was therefore not visible in our case [2].

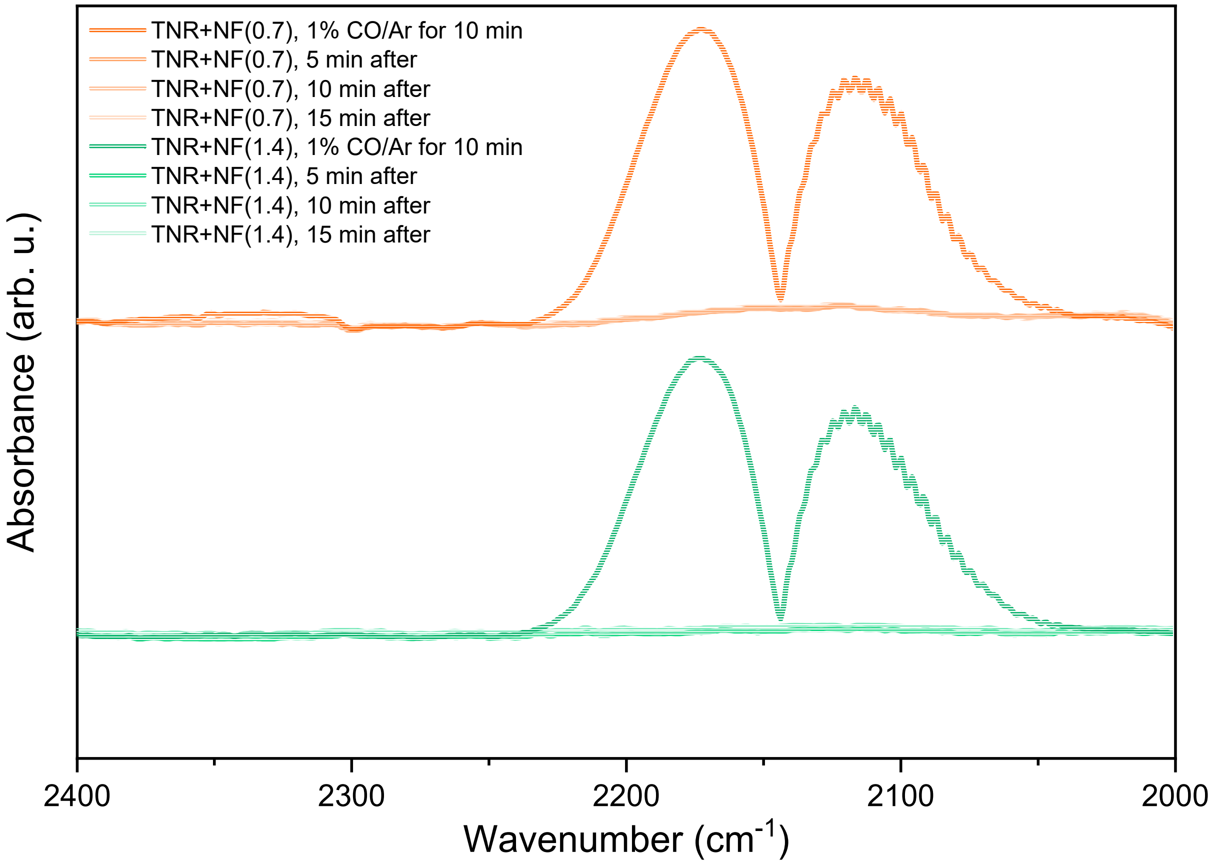

Fig. S7. The results of CO chemisorption measured with DRIFTS analysis. The orange curves show the DRIFTS spectra for the TNR+NF(0.7) sample, after 10 min of purging with 1% CO/Ar and after 5, 10 and 15 min of subsequent purging with N_2. The green curves show the results for the TNR+NF(1.4) sample using the same protocol.

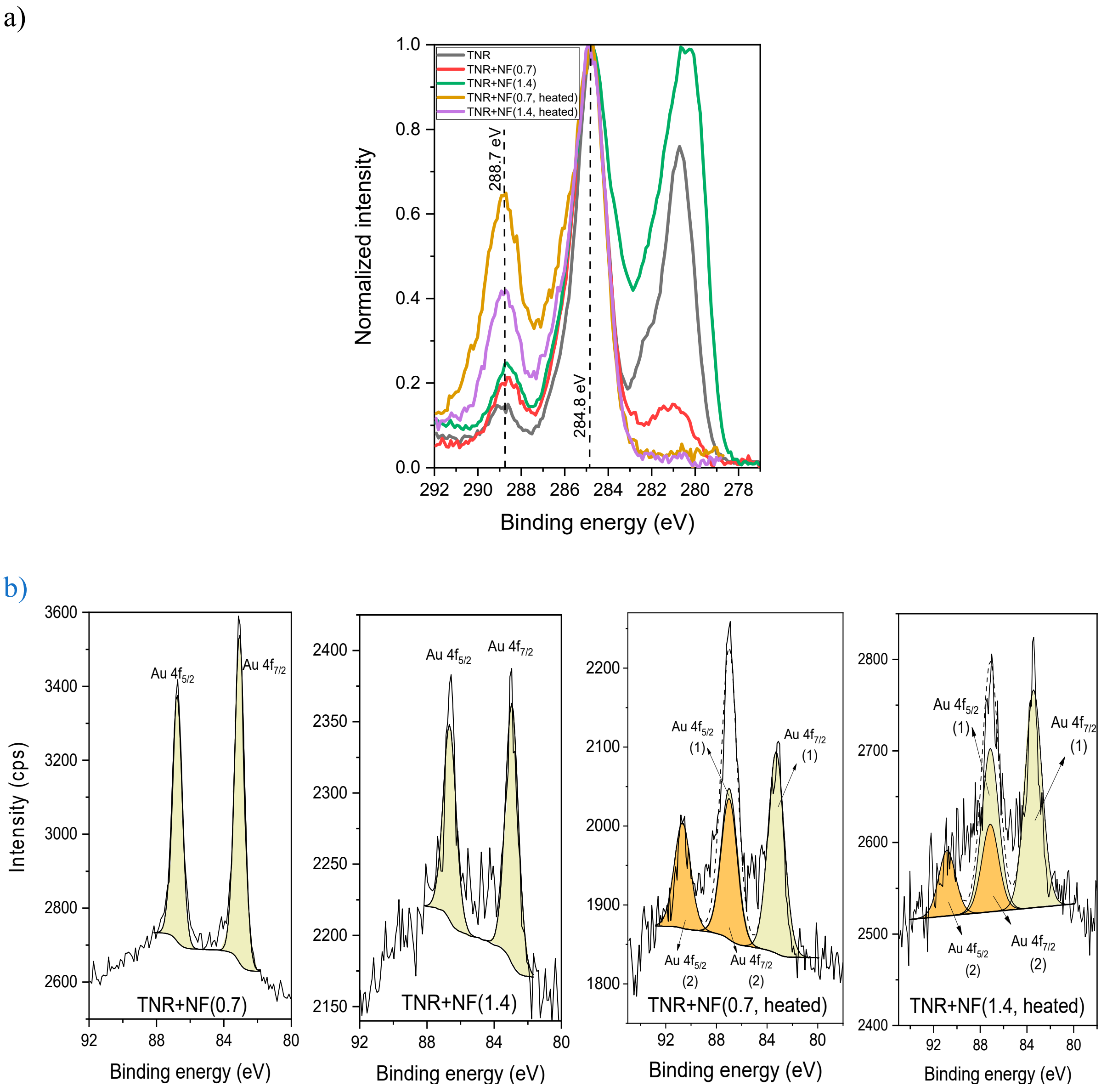

Fig. S8. a) High-resolution C 1s spectra for TNR, TNR+NF(0.7), TNR+NF(1.4), TNR+NF(0.7, heated), and TNR+NF(1.4, heated) samples and b) fitted Au 4f spectra.

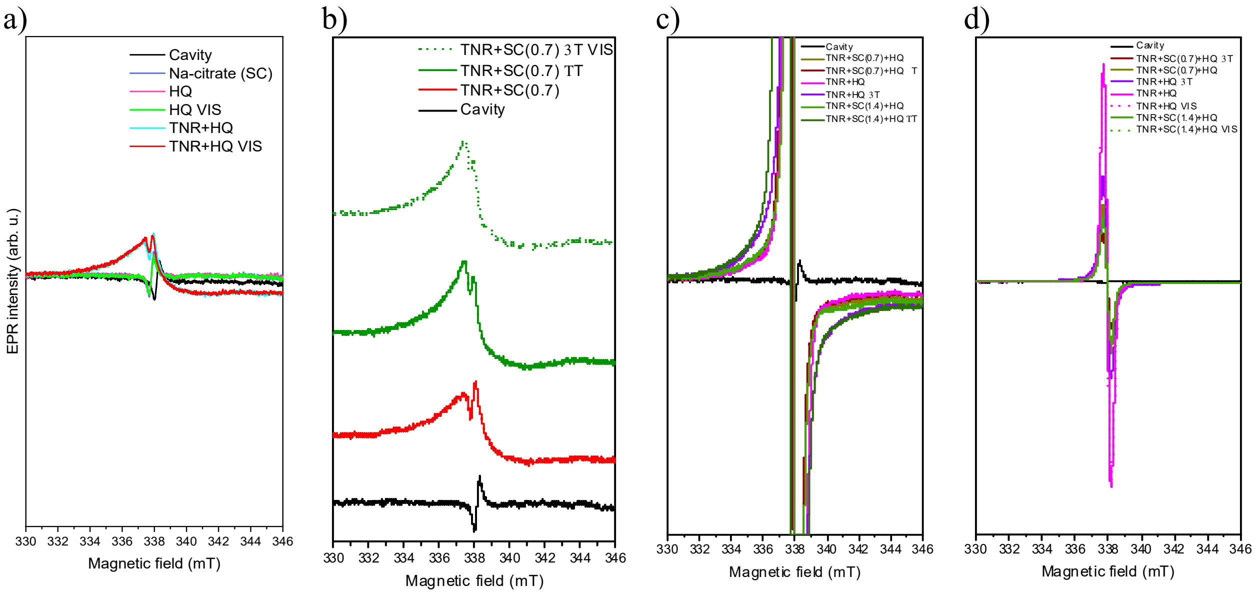

Fig. S9. Solid-state EPR spectra obtained at RT for the synthesis components: pure Na-citrate (SC), hydroquinone (HQ) and a physical mixture of TNR and HQ (a). Figs. S9b and S9c contain EPR spectra measured at RT for samples of synthesis components prepared according to the synthesis procedure, except that an equivalent amount of NaCl was used instead of the gold precursor. Fig. S9d contains the results of the measurements with adjusted instrument parameters (gain factor of 5×10^{1}) for the samples in Fig. S9c. In all cases, visible-light (VIS) irradiation was accumulated for 10 min before the EPR spectra were recorded. ΔT in the sample designation means that the sample was heated at 300 °C in an inert atmosphere prior to the measurements.

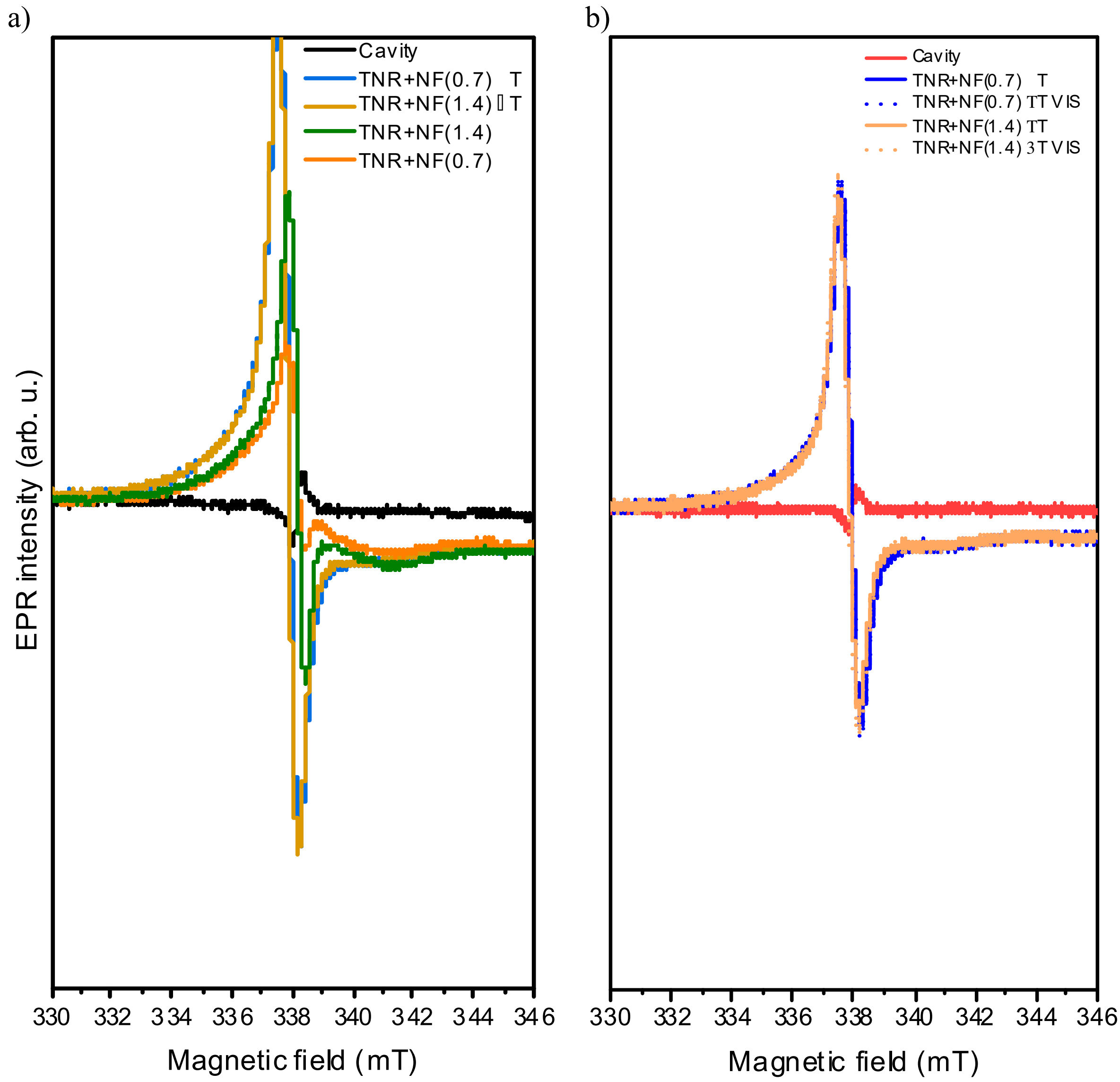

Fig. S10. Results of EPR analysis of heated and unheated catalyst samples measured in darkness and visible light. The gain value for Fig. S10b was 6×10^2 for all samples to obtain the intensity of the EPR spectra within the detector limits. In all cases, visible-light (VIS) irradiation was accumulated for 10 minutes before recording the EPR spectra. ΔT in the sample designation means that the sample was heated at 300 °C in an inert atmosphere prior to the measurements.

a)

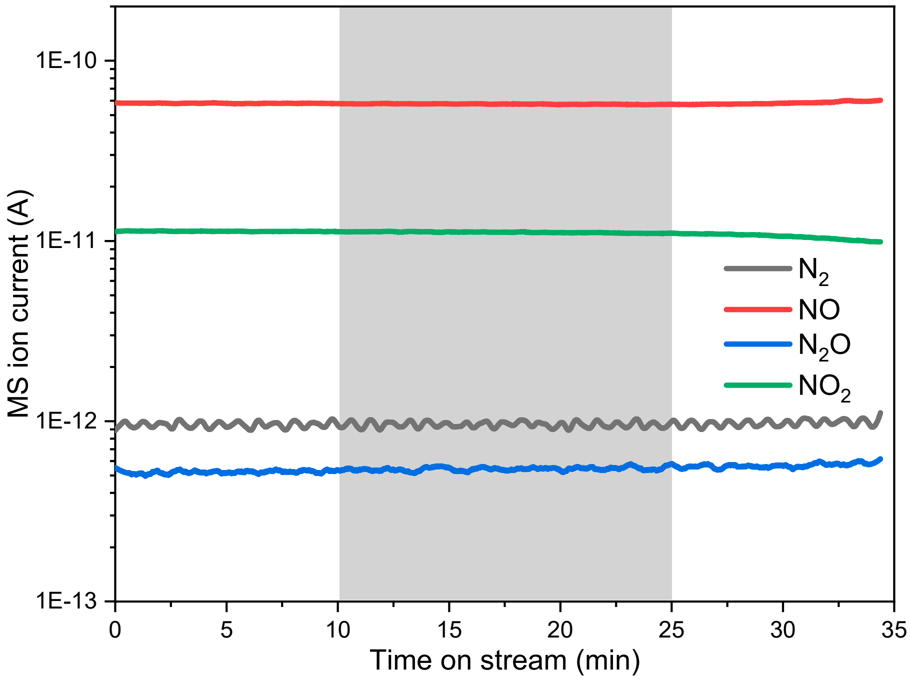

b)

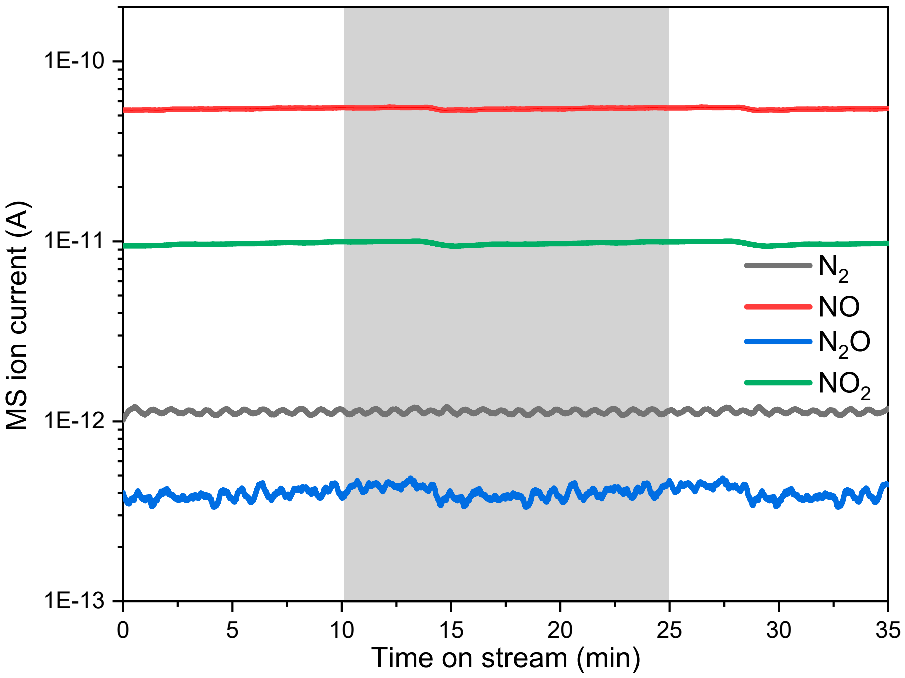

Fig. S11. NO_2, N_2, NO and N_2O mass spectrometer measurements for a) the empty reactor and b) the bare TNR. The reaction took place in a mixture of 5000 ppm NO_2 and 5% H_2 in Ar with a gas flow of 70 ml/min. The grey area shows the time during which the illumination with visible light was switched on.

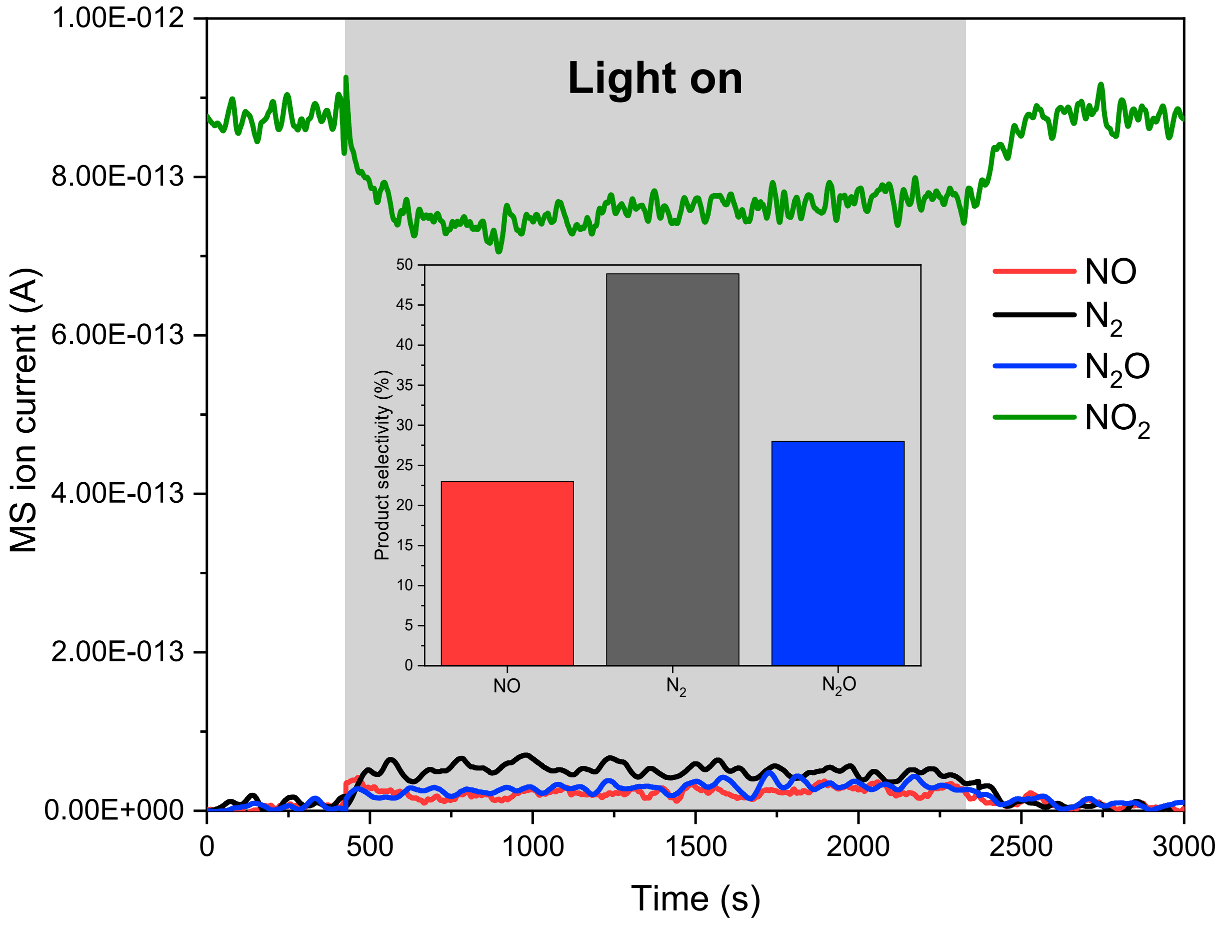

Fig. S12. Time and visible-light illumination dependent NO_2, NO, N_2O and N_2 MS ion current readings for the TNR+NF(0.7) sample to calculate the relative percent selectivity of the three main products (NO, N_2O and N_2) during the visible-light illumination interval (grey area). The results of the calculation are shown in the inset. The reaction was carried out in a mixture of 5000 ppm NO_2 and 5% H_2 in Ar carrier gas at a flow rate of 70 ml/min and 30 °C. The catalyst was exposed to the reaction mixture for 1 hour before being irradiated with visible light.

a)

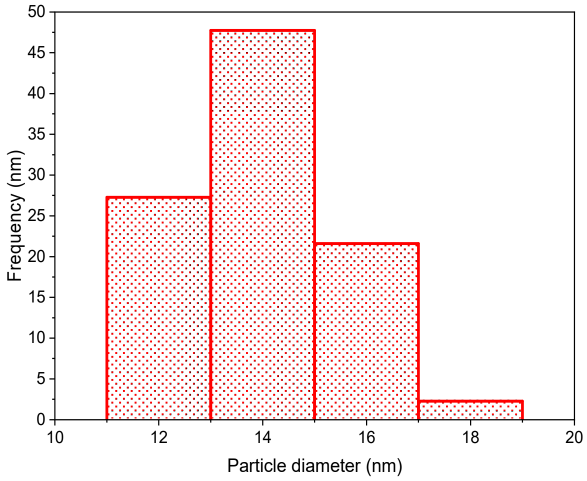

b)

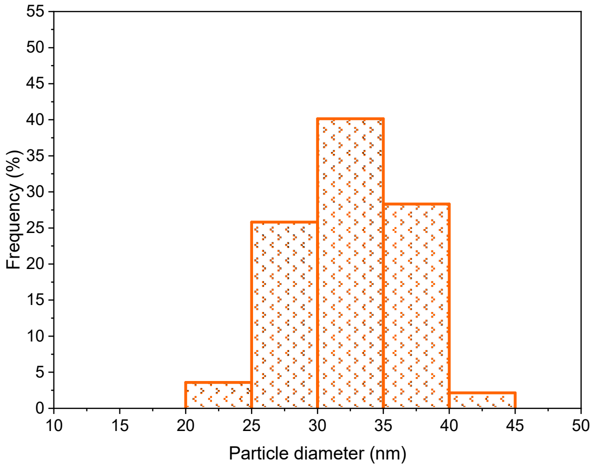

c)

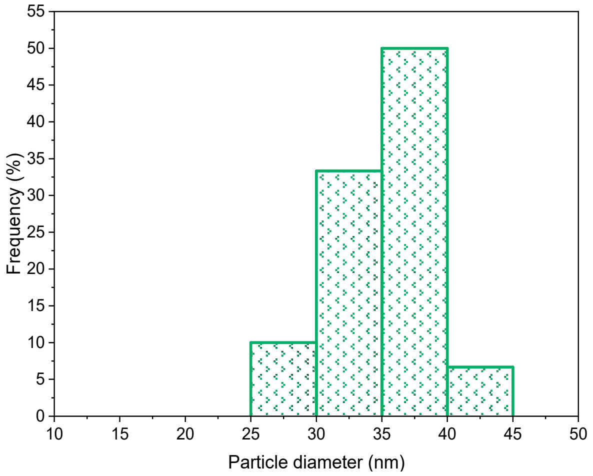

Fig. S13. The results of the size distribution of a) Au-seeds and Au NFs in suspension; b) NF(0.7) and c) NF(1.4) (80-100 measured Au NPs with TEM analysis).

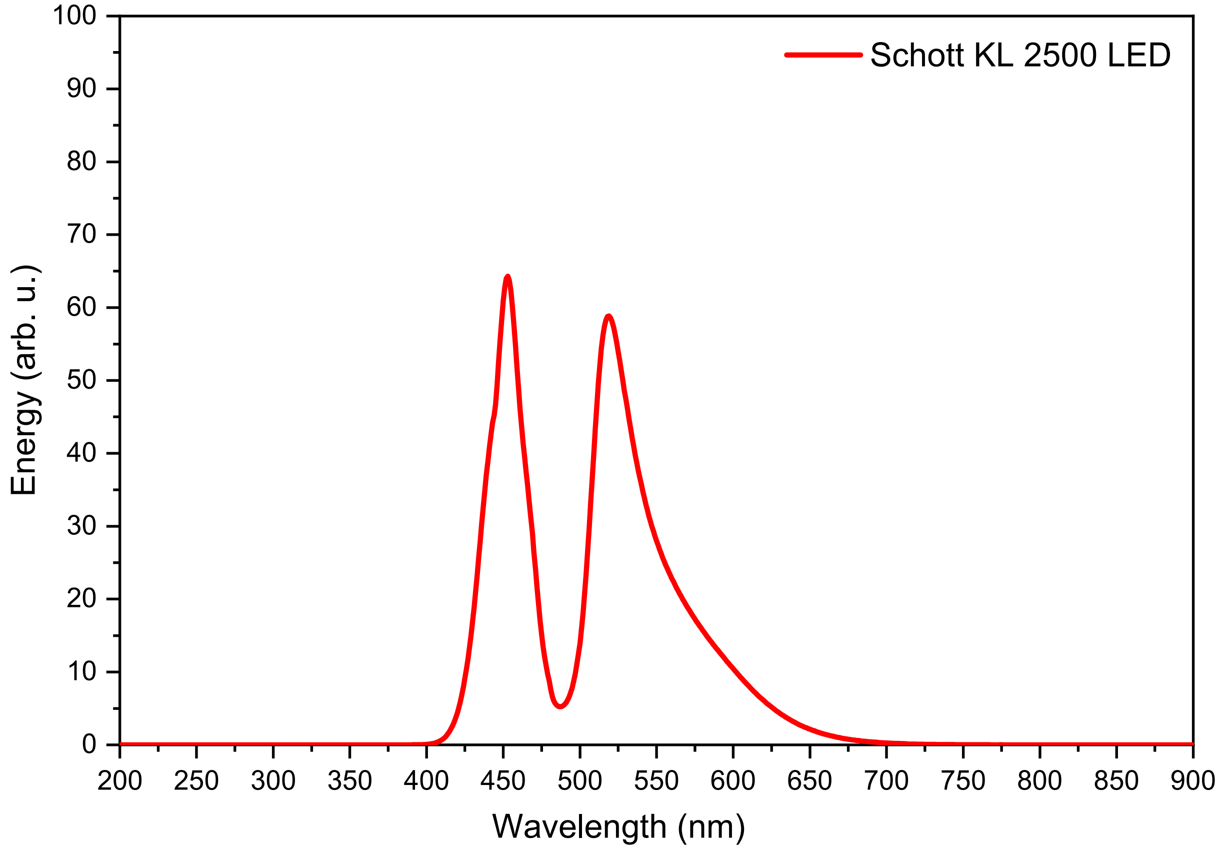

Fig. S14. The energy spectrum of the Schott KL 2500 LED light source used in the process of H_2-assisted NO_2 photocatalytic reduction.

Presence of plasmonic effects in TNR+NF(0.7) catalyst under visible-light illumination

To confirm the proof of concept for the presence of plasmonic effects in the investigated Au/TiO_2 catalysts under visible-light illumination, we used a UV-Vis spectrophotometer (Perkin Elmer, model Lambda 45) as a light source to illuminate a reaction suspension with visible light of a defined wavelength. COUM aqueous solution (200 mg L^{-1}) was used to monitor the ability of TNR+NF(0.7) sample to produce $OH^{\bullet}$ radicals (via H_2O_2 generated from superoxide anion radicals). Oxidation of coumarin with $OH^{\bullet}$ radicals leads to the formation of 7-hydroxycoumarin (7-OHC) as a fluorescent compound [3].

3 mL of the prepared COUM solution and 2 mg of a catalyst TNR+NF(0.7) were mixed in a cuvette for 24 h (800 rpm). The mixture was illuminated with a specific wavelength (541 nm). This wavelength was selected according to the position of the peak in the recorded UV-Vis DR spectrum. The results of the COUM test reaction showed that the concentration of 7-OHC formed in the presence of sample TNR+NF(0.7) was $c_{7\text{-OHC}}=1.36\times10^{-3}$ µM. Slapničar et al. [4] obtained a result of $c_{7\text{-OHC}}=3.72\times10^{-4}$ µM in their study with spherical Au NPs of similar size, which confirms that nanoflowers have a more intense plasmonic effect than spherical NPs. The obtained result is an important confirmation of the presence of a plasmonic effect in the investigated TNR+NF(0.7) catalyst.

Table S1

Results of SEM-EDXS analysis of the investigated TNR+NF(1.4) catalyst.

Sample		TNR+NF(1.4), bottom layer	TNR+NF(1.4), top layer
Ti	wt.%	47.1±0.3	56.0±0.3
O		52.5±0.3	42.8±0.3
Au		0.4±0.1	1.2±0.2

Table S2

Calculated g-values from solid-state EPR spectra of investigated photocatalysts for P1+P3, P2 and P4 signals. Samples marked with LN2 were measured at the temperature of liquid nitrogen.

Sample	TNR	TNR+NF(0.7)	TNR+NF(1.4)
P1+P3	2.007	2.006	2.006
P2	1.986	1.986	1.985
P4	ND	2.003	2.004
$P1+P3^{LN2}$	2.007	2.005	2.005
$P2^{LN2}$	1.997	1.997	ND
$P4^{LN2}$	ND	2.003	2.003

ND – Not detected.

References

1. Boccuzzi, F.; Chiorino, A.; Chemisorption and catalytic properties of gold nanoparticles on different oxides: electronic or structural effects? *Stud. Surf. Sci. Catal.* **2001**, 140, 77–86, https://doi.org/10.1016/S0167-2991(01)80138-5.

2. Hartshorn, H.; Purshell, C.J.; Chandler, B.D.; Adsorption of CO on Supported Gold Nanoparticle Catalysts: A Comparative Study. *J. Phys. Chem. C* **2009**, 113, 10718–10725, https://doi.org/10.1021/jp902553n.

3. Žerjav, G.; Albreht, A.; Vovk, I.; Pintar, A.; Revisiting terephthalic acid and coumarin as probes for photoluminescent determination of hydroxyl radical formation rate in heterogeneous photocatalysis. *Appl. Catal. A* **2021**, 598, 117566, https://doi.org/10.1016/j.apcata.2020.117566.

4. Slapničar, Š.; Žerjav, G.; Zavašnik, J.; Finšgar, M.; Pintar, A.; Synthesis and characterization of plasmonic Au/TiO_2 nanorod solids for heterogeneous photocatalysis. *J. Environ. Chem. Eng.* **2023**, 11, 109835, https://doi.org/10.1016/j.jece.2023.109835.
